# Efficacy and safety of dihydroartemisinin–piperaquine versus artemether–lumefantrine for treatment of uncomplicated *Plasmodium falciparum* malaria in Ugandan children: a systematic review and meta-analysis of randomized control trials

**DOI:** 10.1186/s12936-021-03711-4

**Published:** 2021-04-01

**Authors:** Dawit Getachew Assefa, Eden Dagnachew Zeleke, Delayehu Bekele, Hanna Amanuel Tesfahunei, Emnet Getachew, Michele Joseph, Tsegahun Manyazewal

**Affiliations:** 1 College of Health Sciences, Center for Innovative Drug Development and Therapeutic Trials for Africa (CDT-Africa), Addis Ababa University, P.O. Box 9086, Addis Ababa, Ethiopia; 2 Department of Nursing, College of Health Science and Medicine, Dilla University, Dilla, Ethiopia; 3 Department of Midwifery, College of Health Science, Bule-Hora University, Bule-Hora, Ethiopia; 4 Department of Obstetrics and Gynecology, Saint Paul’s Hospital Millennium Medical College, Addis Ababa, Ethiopia; 5 Hager Biomedical Research Institute, Asmara, Eritrea; 6 Arsi University, Asella, Ethiopia

**Keywords:** Uncomplicated *Plasmodium falciparum*, Children, Randomized controlled trial, Artemisinin combination therapies, Dihydroartemisinin–piperaquine, Artemether–lumefantrine, Systematic review and meta-analysis, Uganda

## Abstract

**Background:**

The emergence of artemisinin resistance in Southeast Asia and *Plasmodium falciparum kelch13* propeller gene mutations in sub-Saharan African pose the greatest threat to global efforts to control malaria. This is a critical concern in Uganda, where artemisinin-based combination therapy (ACT) is the first-line treatment for uncomplicated falciparum. The objective of this study was to compare the efficacy and safety of dihydroartemisinin–piperaquine (DHA–PQ) and artemether–lumefantrine (AL) for the treatment of uncomplicated falciparum malaria in Ugandan children.

**Methods:**

A search of PubMed and the Cochrane Central Register of Controlled Trials for retrieving randomized controlled trials comparing the efficacy and safety of DHA–PQ and AL for treatment of uncomplicated falciparum malaria in Ugandan children was done. The search was performed up to 31 August 2020. The data extracted from eligible studies and pooled as risk ratio (RR) with a 95% confidence interval (CI), using Rev Man Software (5.4). The protocol was registered in PROSPERO, ID: CRD42020182354.

**Results:**

Eleven trials were included in this review and two of them only included under safety outcome. Total 3798 participants were enrolled. The PCR unadjusted treatment failure was significantly lower with DHA–PQ at day 28 (RR 0.30, 95% CI 0.19–0.49; participants = 7863; studies = 5; I^2^ = 93%, low quality evidence) and at day 42 (RR 0.53, 95% CI 0.38–0.76; participants = 1618; studies = 4; I^2^ = 79%, moderate quality of evidence). The PCR adjusted treatment failure at day 42 was significantly lower with DHA–PQ treatment group (RR 0.45, 95% CI 0.28 to 0.72; participants = 1370; studies = 5, high quality of evidence), and it was below 5% in both arms at day 28 (moderate quality of evidence). AL showed a longer prophylactic effect on new infections which may last for up to 63 days (PCR-adjusted treatment failure: RR 2.04, 95% CI 1.13–3.70; participants = 1311; studies = 2, moderate quality of evidence). Compared to AL, DHA–PQ was associated with a slightly higher frequency of cough (RR 1.07, 95% CI 1.01 to 1.13; 2575 participants; six studies; high quality of evidence). In both treatment groups, the risk of recurrent parasitaemia due to possible recrudescence was less than 5% at day 28. The appearance of gametocyte between 29 and 42 days was also significantly lower in DHA–PQ than AL (RR 0.26, 95% CI 0.12 to 0.56; participants = 623; studies = 2; I^2^ = 0%).

**Conclusion:**

Compared to AL, DHA–PQ appeared to reduce treatment failure and gametocyte carriage in Ugandan children. This may trigger DHA–PQ to become the first-line treatment option. Both treatments were safe and well-tolerated.

**Supplementary Information:**

The online version contains supplementary material available at 10.1186/s12936-021-03711-4.

## Background

Malaria remains the major cause of mortality and morbidity in sub-Saharan Africa. According to the 2019 World Malaria report, there were 228 million cases and 405,000 deaths due to malaria in 2018, where 93% of cases and 94% of deaths were from Africa [1–3]. Children aged under 5 years were at high risk of malaria infection, with 24 million children in African infected in 2018 [1]. *Plasmodium falciparum* was the predominant and life-threatening parasite in Africa, causing 99.7% of estimated malaria cases in Africa [3]. Uganda was found to be the home for 16 million malaria cases and 10,500 deaths in 2013 [4]. According to the country’s 2016 national Demographic Health Survey (DHS), the prevalence of malaria had not been reduced nationally and *P. falciparum* remains the species responsible for the vast majority of malaria cases, and the number of malaria cases was increasing in the country, except the West Nile region [5].

Uncomplicated malaria consists of symptoms of malaria and positive parasitological test (microscopy or rapid diagnostic test [RDT]), but with no sign of severe malaria [2, 6]. If it is left untreated, it progresses to severe disease [2], with early diagnosis and treatment playing a crucial role in reducing mortality and morbidity [7]. Since 2004, all malaria-endemic countries have gradually updated their treatment policy from mono-therapy to the currently recommended artemisinin-based combinations [1]. The drug combinations include short-acting artemisinin derivatives, such as artesunate, artemether, or dihydroartemisinin, in combination with long-acting drugs. The artemisinin component covers two asexual cycles and rapidly decrease parasitaemia by a factor of approximately 10,000 in each 48-h asexual cycle. It is also active against the sexual stages that facilitate forward transmission to mosquitoes. Over several weeks after treatment, the partner drug eliminates residual parasites [6].

While the anti-malarial efficacies of presently endorsed artemisinin-based combinations have been excellent in Africa [8, 9], resistance to ACT in Southeast Asia has become an emerging concern [10]. In 2009, a reduction in parasite clearance rate by 100-fold was reported in western Cambodia, exhibiting artemisinin resistance [10]. Since then, artemisinin resistance has been defined as a parasite clearance half-life of ≥ 5 h cut-off after treatment with ACT or artesunate monotherapy [11]. Slow parasite clearance signifies a “partial” resistance that is articulated only in early-ring-stage parasites [12, 13]. Late parasite clearance following treatment with artemisinins, mediated predominantly by mutations in the *kelch13* (*k13*) gene, was detected in the Greater Mekong Sub-region and dozens of *k13*-propeller mutations have been detected at very low frequency in 18 countries in sub-Saharan Africa [14].

Dihydroartemisinin–piperaquine (DHA–PQ) is a promising artemisinin-based combination recently endorsed by the World Health Organization (WHO) as a potential alternative treatment for uncomplicated malaria in Africa. Several clinical trials have revealed that DHA–PQ is safe and efficacious for treatment of uncomplicated malaria [15–18], but analysis of cardiac adverse events in clinical trials showed that QTc prolongation were reported more frequently in DHA–PQ treated patients than in those treated with comparator anti-malarial [19]. The pharmacokinetics and pharmacodynamics of the combination therapy for adults are well documented; however, there have been inconsistencies of these potential effects in children. The emergence of artemisinin resistance in Southeast Asia and *P. falciparum k13* mutations in sub-Saharan African pose the greatest threat to global efforts to control malaria [10, 11, 13, 20]. This is a critical concern in Uganda, where ACT medicines is the first-line treatment option for uncomplicated falciparum. Additionally, in Uganda and other sub-Saharan African countries, malaria-HIV co-infection is associated with an increased frequency of clinical parasitaemia, increased parasite and viral load, impaired immunity to malaria in children, and impaired anti-malarial drug efficacy [21, 22]. The potential benefits of DHA–PQ over other artemisinin-based combinations [23] make it necessary to investigate this further among children in Uganda. This systematic review and meta-analysis of randomized control trials aimed to synthesize the evidence on the efficacy and safety of DHA–PQ versus AL for the treatment of uncomplicated *P*. *falciparum* malaria in Ugandan children.

## Methods

The protocol for this systematic review and meta-analysis has been registered at the International Prospective Register of Systematic Reviews (PROSPERO) database, ID: CRD42020182354 [24]. The Preferred Reporting Items for Systematic Review and Meta-Analysis (PRISMA 2015) guidelines [25] was followed to choose studies to be included in this review.

### Eligibility criteria

The studies included were randomized controlled trials conducted in Uganda that evaluated the efficacy and safety of DHA–PQ versus AL for treatment of uncomplicated falciparum malaria in children, written in English and published between 01 January 2004 to 31 August 2020. Eligible studies were identified through the PICOS format [26].

### Participants

Children having uncomplicated falciparum malaria residing in Uganda, regardless of gender, were included.

### Interventions

A target dose (range) of 4 (2–10) mg/kg bw per day dihydroartemisinin and 18 (16–27) mg/kg bw per day piperaquine given once a day for 3 days for adults and children weighing ≥ 25 kg. The target doses and ranges for children weighing < 25 kg are 4 (2.5–10) mg/kg bw per day dihydroartemisinin and 24 (20–32) mg/kg bw per day piperaquine once a day for 3 days.

### Comparator

A total dose of 5–24 mg/kg bw of artemether and 29–144 mg/kg bw of lumefantrine. Artemether + lumefantrine is given twice a day for 3 days (total, six doses). The first two doses should, ideally, be given 8 h apart.

### Outcome measures

#### Primary outcomes

The treatment outcome was determined according to the classification of WHO Methods and techniques for clinical trials on antimalarial drug efficacy: genotyping to identify parasite populations [27] classified as:

Early treatment failure (ETF): The development of danger signs or severe malaria on days 1, 2, or 3 in the presence of parasitaemia; or parasitaemia on day 2 higher than on day 0; or parasitaemia and axillary temperature > 37.5 °C on day three; or parasitaemia on day 3 > 20% of count on day 0 or development of danger signs, or severe malaria, after day 3 with parasitaemia; or presence of *P. falciparum* parasitaemia and axillary temperature > 37.5 °C on or after day 4; or presence of *P. falciparum* parasitaemia after day 7.

Late clinical failure (LCF): Danger signs or severe malaria in the presence of parasitaemia on any day between day 4 and day 28 (day 42) in patients who did not previously meet any of the criteria for early treatment failure; or Presence of parasitaemia on any day between day 4 and day 28 (day 42) with axillary temperature ≥ 37.5 °C in patients who did not previously meet any of the criteria for early treatment failure.

Late parasitological failure (LPF): Presence of parasitaemia on any day between day 7 and day 28 (day 42) with axillary temperature < 37.5 °C in patients who did not previously meet any of the criteria for early treatment failure or late clinical failure.

Adequate clinical and parasitological response (ACPR): Before and after Polymerase Chain Reaction (PCR) correction used to show the treatment success and it defined as absence of parasitaemia by the end of treatment (day 28) regardless of axillary temperature without previously meeting any of the benchmarks of early treatment failure or late clinical failure or late parasite logical failure.

PCR genotyping was used to define treatment failure corresponding to current WHO recommendations [27]. Adverse events including serious adverse events were also assessed.

PCR-unadjusted total failure (*P. falciparum*): Was calculated as the sum of late treatment failures and early treatment failures (without PCR adjustment). The denominator was excluding participants who did not satisfy the inclusion criteria after randomization and those outcomes not available (for example, those who were lost to follow-up, withdrew consent, other species infection, took another anti-malarial, or failed to complete treatment).

PCR-adjusted total failure (*P. falciparum*): Was calculated as the sum of early treatment failures plus late treatment failures due to PCR-confirmed recrudescence. Participants with indeterminate PCR results, missing PCR results or PCR-confirmed new infections were measured to be involuntary withdrawals and excluded them from the calculation. The denominator excludes participants who did not satisfy the inclusion criteria after randomization, participants with (falciparum reinfection, other species mixed with falciparum reinfection, and undetermined or missing PCR) and those participants for whom an outcome was not available (for example, those who were lost to follow-up, withdrew consent, Other species infection, took another anti-malarial, or failed to complete treatment).

#### Secondary outcomes

Fever clearance: The proportion of patients febrile on each day within 3 days.

Parasite clearance: The proportion of patients clear of parasites on each day within 3 days,

Gametocyte carriage at Baseline and Day 14 or 28 or 42, and

Change in serum hemoglobin level from baseline (minimum 28 days and 42 days follow-up) were also evaluated.

### Search strategy

A computerized systematic search method was used to search for articles from PubMed and the Cochrane Central Register of Controlled Trials (CENTRAL). The search was limited to human studies and published in English language until 31 August 2020. Additionally, we searched ClinicalTrials.gov and the WHO International Clinical Trials Registry Platform, and the US Food and Drug Administration (FDA) to search and assess ongoing or unpublished trials.

The search strategies in PubMed for the MeSH terms and text words was 'uncomplicated malaria in children' [MeSH Terms] OR 'uncomplicated *Plasmodium falciparum* malaria in children' [MeSH Terms] OR 'falciparum malaria in children' [MeSH Terms] OR 'asymptomatic malaria in children' [MeSH Terms] AND 'artemisinin based combination therapy' [MeSH Terms] OR 'artemisinin' [MeSH Terms] OR 'artemether lumefantrine' [MeSH Terms] OR 'coartem' [MeSH Terms] OR 'dihydroartemisinin piperaquine' [MeSH Terms] OR 'Duocotecxin' [MeSH Terms] OR 'Eurartesim' [MeSH Terms] OR 'D-Artepp' [MeSH Terms])) AND 'randomized controlled trial' [MeSH Terms] OR 'controlled clinical trial' [MeSH Terms] OR 'randomized' [MeSH Terms] OR 'drug therapy'[MeSH Terms] OR 'trial' [MeSH Terms] OR 'groups' [MeSH Terms] OR 'humans' [MeSH Terms]).

### Study selection, data collection, and data analysis

The Cochrane Handbook for Systematic Reviews of Interventions [28], the RevMan 5.4 software, and the EndNote X7 were used for data management and analysis. Two authors independently reviewed the results and disagreements resolved through discussion. When clarification was necessary, the trial authors were contacted.

### Data extraction and management

The title and abstract were produced from the electronic search and independently screened by two authors based on RCTs that were assessed human *falciparum* malaria. The information collected were trial characteristics including methods, participants, interventions, and outcomes as well as data on dose and drug ratios of the combinations. Relevant information such as title, name of the journal, year of publication, publication status, study design, study setting, follow-up period, sample size, funding source, baseline characteristics of study subjects, fever clearance, parasite clearance, treatment failure, and gametocyte carriage were extracted from each article using a structured data extraction format adapted from Cochrane. The number of participants randomized and the number analysed in each treatment group for each outcome were also captured. Two authors independently extracted the data and cross-checked. For dichotomous outcomes, the number of participants experiencing the event and the number of participants in each treatment group were documented. For continuous outcomes, the arithmetic means and standard deviations for each treatment group collectively with the numbers of participants in each group were extracted.

### Assessment of risk of bias in included studies

The risk of bias for each trial was evaluated by two authors independently using the Cochrane Collaboration's tool for assessing the 'Risk of bias' [26]. The risks were classified as high risk, unclear risk, and low risk.

### Measures of treatment effect

The main outcomes in this review were total treatment failure at days 28, 42, and 63; PCR-adjusted and PCR unadjusted. Dichotomous data were combined and presented using risk ratios. Continuous data were summarized by arithmetic means and standard deviations, and then data were combined using mean differences. Risk ratios and mean differences were accompanied by 95% CIs. In the forest plot, the upper and the bottom tips of the diamond (the centre of the diamond) represents point estimate and the left and right tips of the diamond represents confidence interval. Also, the treatment arm is on the left side and the one in the right side is comparator arm.

### Assessment of heterogeneity

Heterogeneity among trials was assessed by inspecting the forest plots (to detect overlapping CI) and the Cochrane Q and I^2^ statistic were used to measure heterogeneity among the trials in each analysis, the Chi^2^ test with a P < 0.10 to indicate statistical significance was used, and the results were interpreted following Cochrane Handbook for Systematic Reviews of Interventions Version 6.0, Chapter 10: Analyzing data and undertaking meta-analyses [29]. When substantial heterogeneity (I^2^ > 50%) was identified, it was reported, and explored the possible causes by subgroup analyses.

### Data synthesis

The meta-analyses were done consistent with the recommendations of Cochrane [28]. To aid interpretation, included trials were given identity codes including the first author and the year of publication. Trials were enumerated in forest plots in chronological order of the year the trials were published. A random-effects model was used, as trials were done by different researchers, operating independently, and it could be implausible that all the trials had functionally equivalent, with a common effect estimate.

### Subgroup analysis and investigation of heterogeneity

The potential sources of heterogeneity were investigated through the following subgroup analyses: the known studies with HIV negative participants were compared to studies with both HIV negative and positive participants in the overall assessment because HIV infection has an effect of parasite clearance [30].

### Sensitivity analysis

Studies only with low risk of bias were included and to assess the small study effect, the fixed-effect and random-effect estimates of the intervention were compared.

### Quality of evidence

Quality of evidence was assessed using GRADE criteria and the GRADE pro software [31]. The results were presented in a ‘Summary of Findings’ table. Randomized trials are initially categorized as high quality but downgraded after assessment of five criteria [32]. The levels of evidence were defined as 'high', 'moderate', 'low', or 'very low'. The recommendations of Section 8.5 and Chapter 13 of the Cochrane Handbook for Systematic Reviews of Interventions was followed [33]. The imprecision was judged based on the optimal information size criteria and CI [34].

## Results

### Search results

**Fig. 1:**
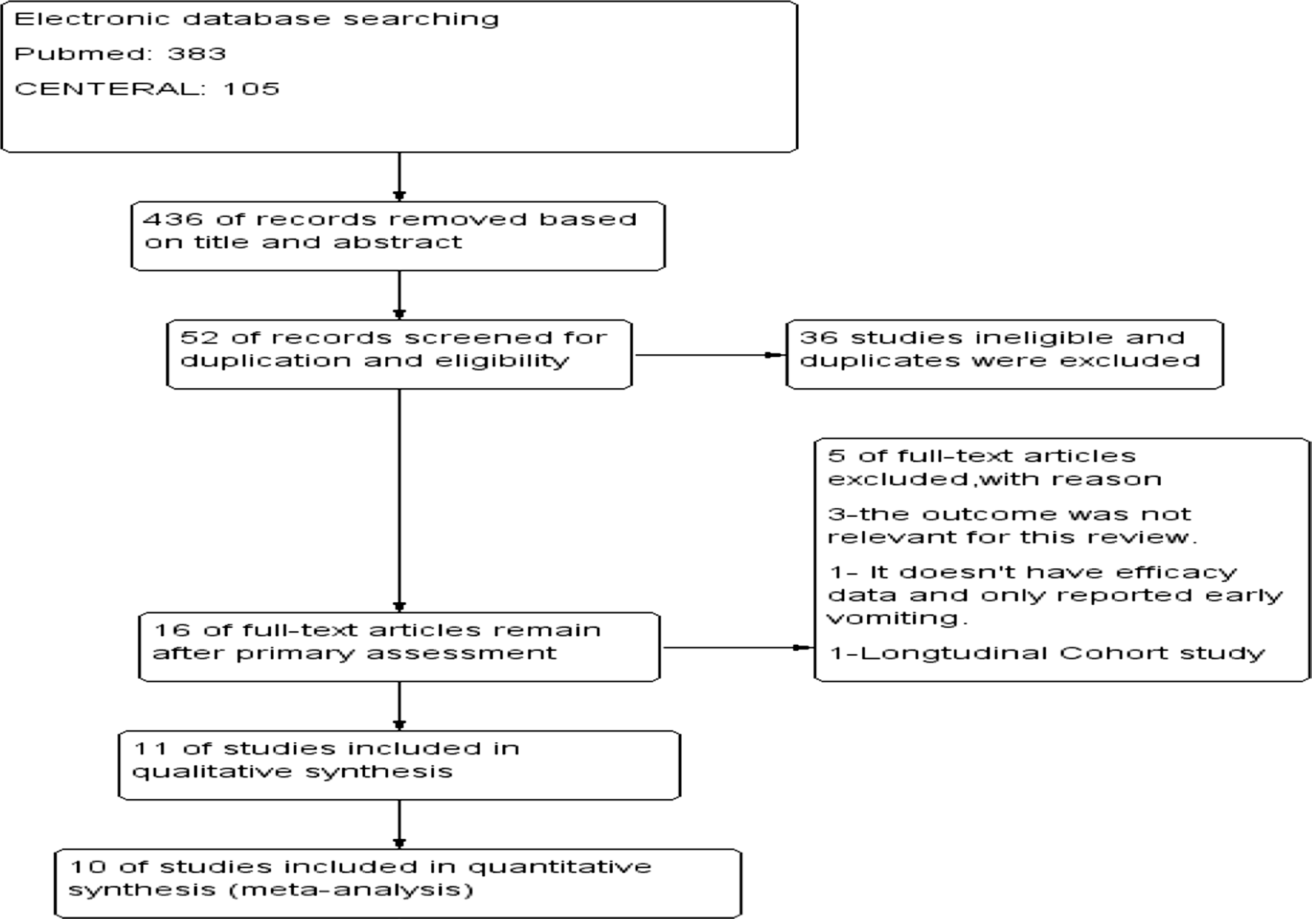
PRISMA study flow diagram of the study

A total of 488 trials through the databases were searched, of which 52 full-text trials for eligibility were assessed and found 10 of them fulfilled the inclusion criteria for meta-analysis and an additional one for qualitative analysis (Fig. 1).

### Study characteristics

In this review, 11 trials were included, which enrolled 3981 participants with uncomplicated *P. falciparum* malaria were included in this review (Table 1).

**Table 1:** Characteristics of included studies

S. no.	Author, Publication year	Study design	Study setting and period	Follow up	Subjects			Patient important outcome	DHA–PQ	AL
					Number of participants		Inclusion age			
					DHA–PQ	AL				
1	Kamya, 2007 [39]	Single blind RCT	Rural health center, March, 2006–July, 2006	42 days	253	256	6 months–10 years	Loss to follow up	0	0
								ETF	0	0
								LCF	19	30
								LPF	73	89
								ACPR	117	89
								Fever clearance at day 1	137	137
								Fever clearance at day 2	66	72
								Fever clearance at day 3	52	57
								Parasite clearance at day 2	1	2
								Parasite clearance at day 3	0	0
								Gametocyte carriage at baseline	9	18
								Gametocyte carriage day 1–14	5	2
								Gametocyte carriage at day 15–28	0	5
								Gametocyte carriage at day 29–41	4	11
								Hgb at baseline mean (SD)	9.5 (1.8)	9.7 (1.9)
								Hgb g/dL at day 42 mean (SD)^a^	1.9 (1.8)	1.5 (1.8)
								Vomiting	65	65
								Diarrhea	25	19
								Anorexia	90	91
								Abdominal pain	19	20
								Weakness/malaise	85	103
								Cough	136	133
								Coryza	127	121
								Pruritus	14	22
								SAE	4	2
2	Yeka, 2008 [40]	Single-blind RCT	Health center, August 2006–April 2007	42 days	234	227	6 months–10 years	Los to follow up	3	3
								ETF	0	1
								LCF	9	23
								LPF	17	41
								ACPR	186	131
								Fever clearance at day 1	117	133
								Fever clearance at day 2	44	37
								Fever clearance at day 3	22	22
								Parasite clearance at day 2	7	5
								Parasite clearance at day 3	0	0
								Gametocyte carriage at baseline	12	18
								Gametocyte carriage day 1–14	4	1
								Gametocyte carriage at day 15–28	1	7
								Gametocyte carriage at day 29–41	4	13
								Hgb at baseline mean (SD)	9.9 (2.1)	9.9 (1.8)
								Hgb at day 42 mean (SD)^a^	1.8 (1.8)	1.7 (2.0)
								Vomiting	35	35
								Diarrhea	26	23
								Anorexia	47	49
								Abdominal pain	17	24
								Weakness/malaise	28	27
								Cough	164	150
								Coryza	159	150
								Pruritus	8	3
								SAE	5	2
3	Arinaitwe, 2009 [35]	Open-label RCT	Local antenatal clinics in Tororo, August 2007–July 2008	63 days	119	111	6 weeks–12 months	Other anti-malaria use	1	2
								Loss to follow up	5	2
								Complicated malaria at day 0	0	1
								Recurrent malaria caused by non-falciparum species	0	1
								ETF	0	0
								LCF	13	45
								LPF	26	64
								ACPR	306	205
								Fever clearance at day 1	138	163
								Fever clearance at day 2	13	17
								Fever clearance at day 3	9	12
								Parasite clearance at day 2	12	22
								Parasite clearance at day 3	1	0
								Gametocyte carriage at baseline	30	26
								Gametocyte carriage day 1–14	10	1
								Gametocyte carriage at day 15–28	1	0
								Hgb at baseline mean (SD)	9.9 (1.5)	9.8 (1.5)
								Hgb at day 28 mean (SD)^a^	0.6 (1.68)	0.6 (1.56)
								Vomiting	23	20
								Diarrhea	79	86
								Anorexia	3	0
								Weakness	1	0
								Cough	177	153
								Pruritus	0	0
								SAE	3	1
4	Bassat, 2009 [76]	Open-label RCT (non-inferiority)	Rural sites and one peri-urban site, August 2005–July 2006	42 days	164	82	6–59 months	PCR-corrected ACPR day 28	155	77
								PCR-corrected ACPCR day 42	154	77
5	Katrak, 2009 [42]	Open-label RCT	N/A, August, 2007–April, 2008	N/A	124	122	6 weeks–12 months	Vomiting	19	9
								Diarrhea	64	76
								Cough	200	164
								SAE	3	2
6	4ABC, 2011 [36]	Open-label RCT	N/A, July 2007–June 2009	63 days	422	421	6–59 months	Day 28 PCR-unadjusted ACPR	360	313
								Day 28 PCR-adjusted ACPR	407	393
								Day 63 PCR-unadjusted ACPR	234	209
								Day 63 PCR-adjusted ACPR	375	371
7	Yeka, 2013 [37]	Single-blind RCT	Health center, December, 2007–April, 2009	28 days	72	35	6–59 months	LTF	0	0
								ETF	0	0
								LCF	6	7
								LPF	12	14
								ACPR	54	14
								Fever clearance at day 1	48	14
								Fever clearance at day 2	15	21
								Fever clearance at day 3	4	2
								Parasite clearance at day 2	2	5
								Parasite clearance at day 3	0	0
								Gametocyte carriage at baseline	0	0
								Gametocyte carriage day 1–14	0	0
								Gametocyte carriage at day 15–28	0	0
								Hgb at baseline mean (SD)	10.8 (1.34)	10.6 (1.41)
								Hgb at day 28 mean (SD)^a^	0.8 (1.7)	0.9 (1.7)
								Vomiting	3	2
								Diarrhea	1	2
								Anorexia	12	3
								Abdominal pain	5	0
								Weakness/malaise	1	0
								Cough	47	20
								SAE	0	0
8	Kakuru, 2014 [77]	RCT	District Hospital, August 2007 and April 2008	N/A	21	22	6 weeks–12 months	Loss to follow up	2	2
								ETF	0	0
								LCF	3	19
								LPF	11	53
								ACPR	149	127
								Fever clearance at day 1	46	106
								Fever clearance at day 2	7	16
								Fever clearance at day 3	5	3
								Parasite clearance at day 2	5	32
								Parasite clearance at day 3	1	3
								Gametocyte carriage at baseline	15	9
								Gametocyte carriage at day 15–28	30	15
								Hgb at baseline mean (SD)	9.6 (1.5)	10.1 (1.4)
								Hgb at day 28 mean (SD)^a^	1.0 (1.4)	0.6 (1.5)
								Vomiting	8	18
								Diarrhea	27	23
								Anorexia	6	4
								Weakness/malaise	2	2
								Cough	64	74
9	Muhindo, 2014 [30]	Open-label, RCT	October District Hospital, October 2011–December, 2012	28 days	106	96	6 weeks–12 months			16
								Loss to follow up	13	
								ETF	1	0
								LCF	7	74
								LPF	22	137
								ACPR	311	189
								Fever clearance at day 1	65	124
								Fever clearance at day 2	11	8
								Fever clearance at day 3	7	7
								Parasite clearance at day 1	181	269
								Parasite clearance at day 2	20	23
								Parasite clearance at day 3	1	0
								Hgb at baseline mean (SD)	11.2 (1.5)	11.1 (1.5)
10	Wanzira, 2014 [38]	Open-label, RCT	District Hospital, February 2009–2012	28 days	154	158	4 weeks–12 months	Other anti-malaria use	3	5
								Loss to follow up	21	19
								Withdrawn consent	1	2
								ETF	2	15
								LCF	48	475
								LPF	182	894
								ACPR	2403	1494
11	Yeka, 2019 [41]	Single-blind RCT	Health center and Hospital, October 2015–December, 2016	42 days	299	300	6–59 months	No outcome	11	10
								ETF	0	0
								LCF	32	50
								LPF	43	85
								ACPR	213	155
								Fever clearance at day 1	208	231
								Fever clearance at day 2	71	72
								Fever clearance at day 3	31	18
								Parasite clearance at day 1	219	245
								Parasite clearance at day 2	22	34
								Parasite clearance at day 3	3	3
								Gametocyte carriage at baseline	59	60
								Gametocyte carriage at day 1–42	43	46
								Hgb at day 42 mean (SD)^a^	1.3 (1.69)	0.8 (1.8)
								Vomiting	56	61
								Diarrhea	155	114
								Anorexia	12	3
								Abdominal pain	41	45
								Headaches	18	24
								Weakness/malaise	42	33
								Cough	233	203
								Pallor	22	13
								Skin rash	56	42
								Pruritus	24	16
								SAE	6	6

*LTF* loss to follow up, *ETF* early treatment failure, *LCT* late clinical failure, *LPF* late parasitological failure, *ACPR* Adequate clinical and parasitological response, *AL* artemether–lumefantrine, *DHA–PQ* dihydroartemisinin–piperaquine, *Hgb* hemoglobin, *SD* standard deviation, *PCR* polymerase chain reaction, *SAE* serious adverse event, *N/A* not available

^a^The mean increase in hemoglobin values from the baseline

### Methodological quality and risk of bias

**Fig. 2:**
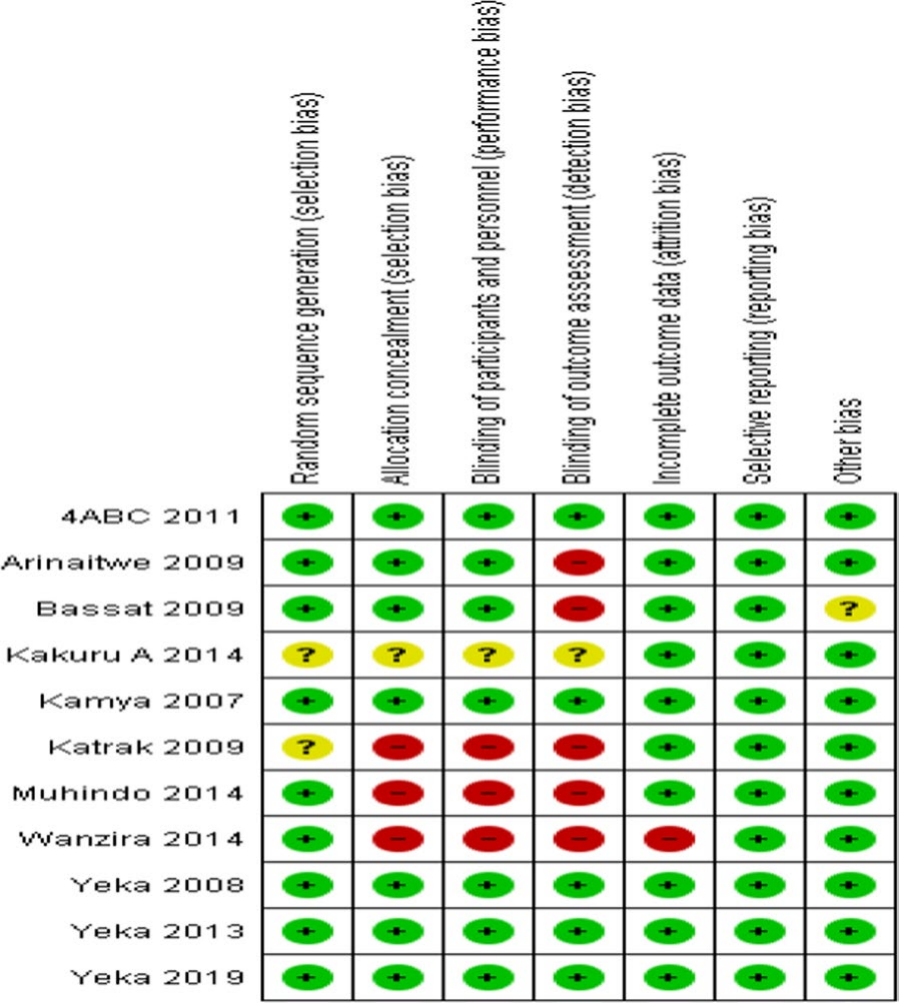
Risk of bias summary: ‘review authors' judgments about each risk of bias item for each included study

The 'Risk of bias' assessments were summarized in Fig. 2.

### Effect of interventions

#### Treatment failure

##### PCR-unadjusted total failure at day 28

**Fig. 3:**
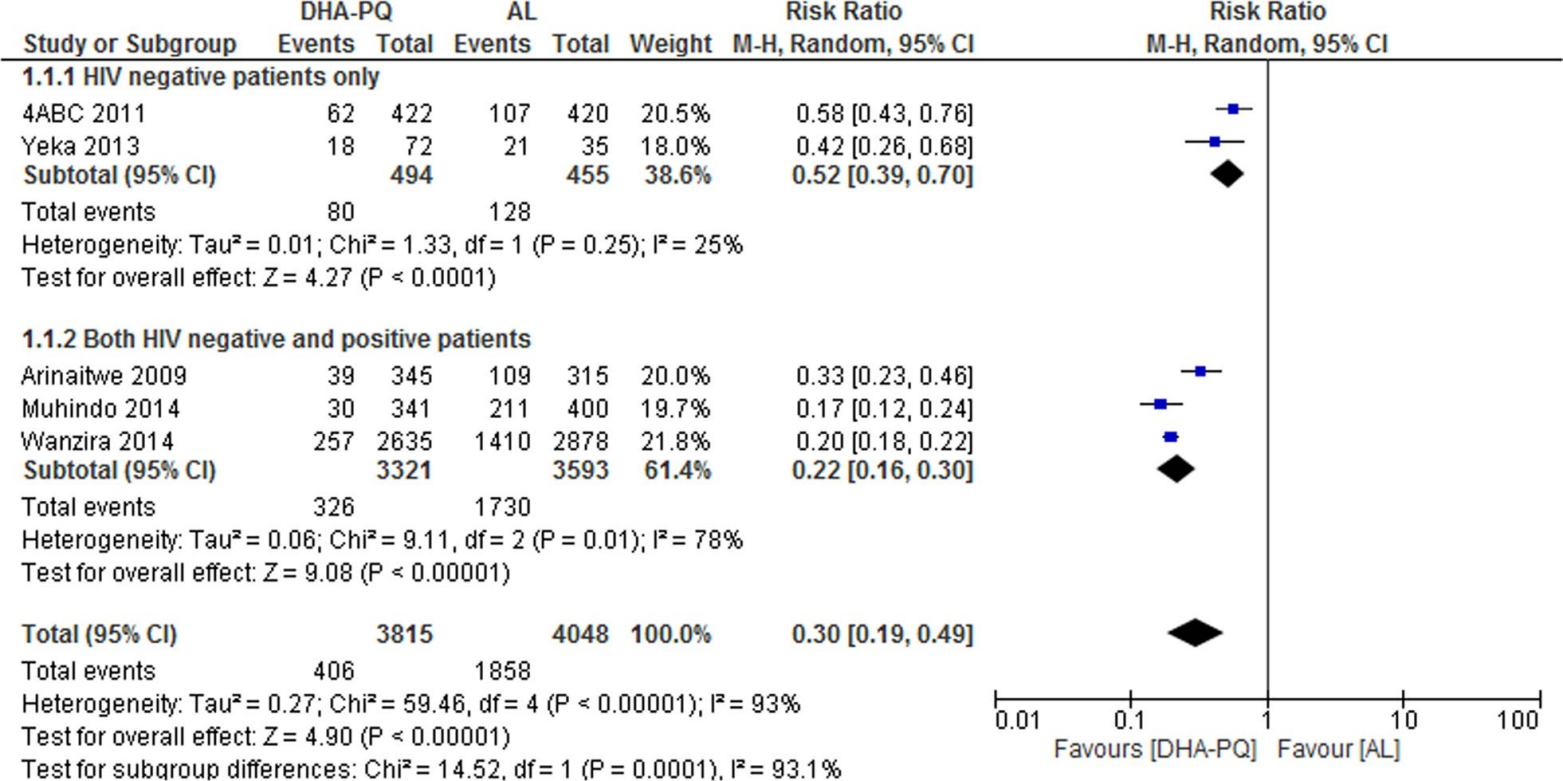
Forest plot of comparison: Dihydroartemisinin piperaquine versus artemether–lumefantrine, outcome: PCR-unadjusted treatment failures at day 28

At day 28, PCR PCR unadjusted treatment failures in five studies [30, 35–38] was significantly lower for participants treated with DHA–PQ than for those treated with AL (RR 0.30, 95% CI 0.19 to 0.49; participants = 7863; studies = 5; I^2^ = 93%, Fig. 3). There was considerable heterogeneity between the studies. To investigate the cause of heterogeneity, the sub-group analyses have done based on the HIV status of the participants in the included studies.

At day 28, in two studies with HIV negative participants, the PCR unadjusted treatment failures was significantly lower for participants treated with DHA–PQ than those treated with AL (RR 0.52, 95% CI 0.39 to 0.70; participants = 949; studies = 2; I^2^ = 25%, Fig. 3).

Consistently, in three studies (participants = 6914; [30, 35, 38]) with both HIV negative and positive participants, the PCR unadjusted treatment failures was significantly lower for participants treated with DHA–PQ than those treated with AL. The results were highly heterogeneous (Heterogeneity: Tau^2^ = 0.06; Chi^2^ = 9.11, df = 2 (P = 0.01); I^2^ = 78%). Relative risks for the individual studies were: 0.33 (95% confidence interval 0.23 to 0.46, [35]); 0.17 (95% confidence interval 0.12 to 0.24, [30]); and 0.20 (95% confidence interval 0.18 to 0.22, [38]). Hence, statistically significant difference was found between the two subgroups (Chi^2^ = 14.52, df = 1 (P = 0.0001), I^2^ = 93.1%, Fig. 3).

##### PCR-adjusted total failure at day 28

**Fig. 4:**
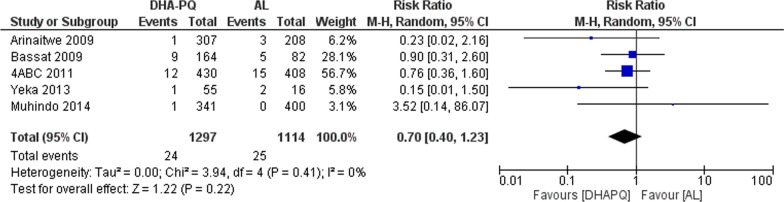
Forest plot of comparison: Dihydroartemisinin piperaquine versus artemether–lumefantrine, outcome: PCR-adjusted treatment failures at day 28

At day 28, the PCR adjusted treatment failures was below 5% in both treatment arms without significant difference between the two treatment groups (RR 0.70, 95% CI 0.40 to 1.23; participants = 2411; studies = 5; I^2^ = 0%, Fig. 4).

##### PCR-unadjusted total failure at day 42

**Fig. 5:**
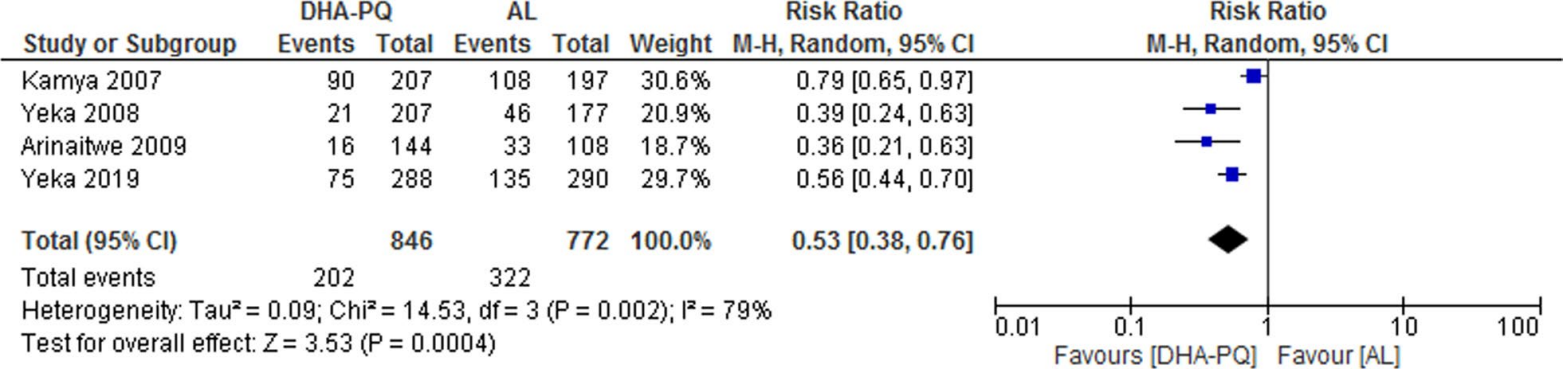
Forest plot of comparison: Dihydroartemisinin–piperaquine versus artemether–lumefantrine, outcome: 1.3 PCR-unadjusted treatment failures at day 42

At day 42, PCR unadjusted treatment failure in four trails [35, 39–41] (participants = 1618) were significantly lower in the DHA–PQ group than the AL group (RR 0.53, 95% CI 0.38 to 0.76; participants = 1618; studies = 4; I^2^ = 79%). The result had considerable heterogeneity and we couldn't pool the result. The PCR unadjusted treatment failures for the individual studies were: 0.79 (95% confidence interval 0.65, 0.97 [39]); 0.39 (95% confidence interval 0.24 to 0.63 [40]); 0.36 (95% confidence interval 0.21, 0.63, [35]) and 0.56 (95% confidence interval 0.44 to 0.70, [41], Fig. 5).

##### PCR-adjusted total failure at day 42

**Fig. 6:**
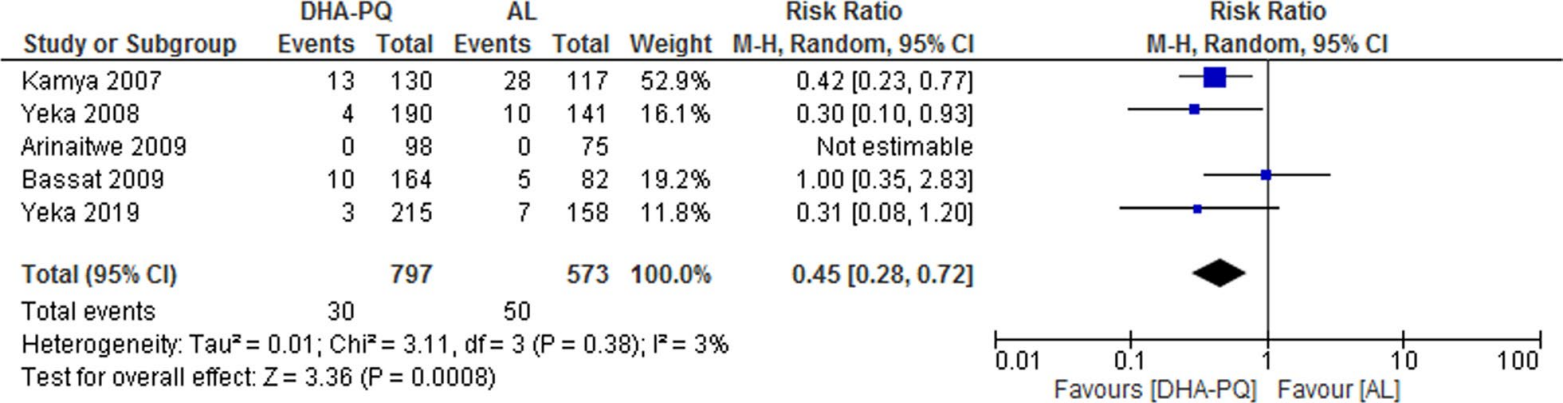
Forest plot of comparison: Dihydroartemisinin piperaquine versus artemether–lumefantrine, outcome: PCR-adjusted treatment failures at day 42

The overall PCR adjusted treatment failures was lower for participants treated with DHA–PQ than those treated with AL (RR 0.45, 95% CI 0.28 to 0.72; participants = 1370; studies = 5; I^2^ = 3%, Fig. 6).

##### PCR-unadjusted total failure at day 63

The PCR unadjusted treatment failure was not statistically different between the two treatment groups (RR 0.59, 95% CI 0.25 to 1.37; participants = 1514; studies = 2; I^2^ = 96%). The result had considerable heterogeneity. It is more useful to consider individual trial results. At day 63, in one study [36] the PCR unadjusted treatment failure in DHA–PQ arm was significantly lower than those treated with AL RR 0.38 (95% confidence interval 0.28 to 0.52) and although, no significant difference was found between the two treatment group in the other trial [35] 0.88 (95% confidence interval 0.77 to 1.02, Additional file 1: S1).

##### PCR-adjusted total failure at day 63

The pooled PCR adjusted treatment failure in participants treated with AL was significantly lower than those who are treated with DHA–PQ (RR 2.04, 95% CI 1.13 to 3.70; participants = 1311; studies = 2; I^2^ = 0%, Additional file 2: S2).

### Fever clearance

#### Fever clearance at Day 1

Six studies with 2978 were reported in this outcome, but the pooled result showed considerable heterogeneity between studies. The result of studies was heterogeneous (RR 0.87, 95% CI 0.75 to 1.01; participants = 2978; studies = 6; I^2^ = 78%). Four trials with HIV negative participants, in two studies [40, 41] the patients treated with DHA–PQ experienced high resolution of fever than AL (RR 0.81 95% CI 0.70 to 0.95 and RR 0.90 95% CI 0.82 to 1.00) and one study [39] no significant difference was found between the two intervention groups (RR 1.00 95% CI 0.87 to 1.14). However, another study [37] reported that the patients treated with AL experienced high resolution of fever (RR 1.67 95% CI 1.08 to 2.58), Additional file 3: S3).

Two studies [30, 35] with both HIV negative and positive participants reported that more participants from DHA–PQ treatment group experienced fast resolution of fever (RR 0.71, 95% CI 0.57 to 0.88; participants = 1441; studies = 2; I^2^ = 51%, Fig. 9). There was statistically significant difference found between the two subgroups (Chi^2^ = 4.91, df = 1 (P = 0.03), I^2^ = 79.6%).

#### Fever clearance at Day 2

By day 2 in five trials, the patients experienced high resolution of fever without a statistically significant difference between the two groups, and in one trial patients treated with DHA–PQ experienced high resolution of fever [37]. The results were highly heterogeneous (RR 0.84, 95% CI 0.61 to 1.16; participants = 2978; studies = 6; I^2^ = 69%). Relative risks for the individual studies were: 0.35 (95% confidence interval 0.21, 0.59 [37]); 0.91 (95% confidence interval 0.69, 1.20 [39]); 1.10 (95% confidence interval 0.74, 1.63 [40]); 0.70 (95% confidence interval 0.34, 1.41 [35]); 1.62 (95% confidence interval 0.66, 3.97 [30]); and 0.99 (95% confidence interval 0.74, 1.32, [41], participants = 2978; studies = 6, Additional file 4: S4).

#### Fever clearance at Day 3

The prevalence of fever was similar over 3 days of follow up in both treatment groups in two trials [37, 39]. The overall pooled result was (RR 1.01, 95% CI 0.80 to 1.27; participants = 2978; studies = 6; I^2^ = 0%, Additional file 5: S5).

### Parasite clearance

The percentage of patients with parasitaemia at day one in two trials was significantly lower in the DHA–PQ treatment group than AL (RR 0.85, 95% CI 0.75 to 0.97; participants = 1369; studies = 2; I^2^ = 66%, Additional file 6: S6). However, at day 2 and 3, the overall result shows that the percentage of patients with parasitaemia was lower in both treatment groups without statistically significant difference (RR 0.69, 95% CI 0.47 to 1.01; participants = 2978; studies = 6; I^2^ = 21, Additional file 6: S6) and (RR 1.46, 95% CI 0.40 to 5.36; participants = 2978; studies = 6; I^2^ = 0%, Additional file 6: S6).

### Gametocytes

#### Gametocyte carriage at baseline

There was no significant difference in the appearance of gametocytes at baseline between two treatment groups (RR 0.71, 95% CI 0.46 to 1.10; participants = 2083; studies = 5; I^2^ = 61%, Additional file 7: S7).

##### Gametocyte carriage

**Fig. 7:**
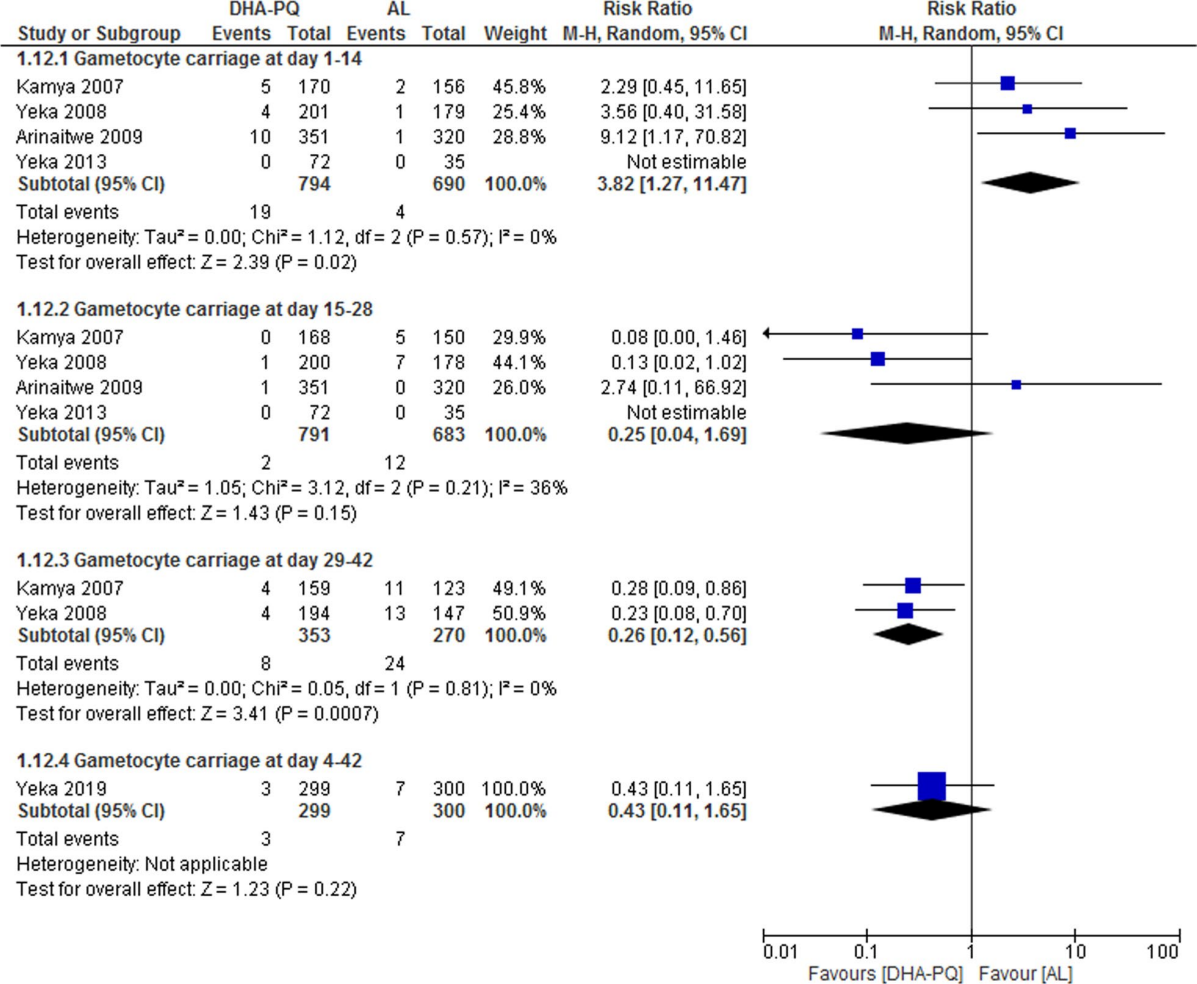
Forest plot of comparison: Dihydroartemisinin–piperaquine versus artemether–lumefantrine, outcome: Gametocyte carriages

The overall gametocyte appearance at day 1–14 and 29–42 was significantly lower in patients treated with AL than DHA–PQ (RR 3.82, 95% CI 1.27 to 11.47; participants = 1484; studies = 4; I^2^ = 0%, Fig. 7) and (RR 0.26, 95% CI 0.12 to 0.56; participants = 623; studies = 2; I^2^ = 0%, Fig. 7). However, at day 15–28, the appearance of gametocyte carriage was lower in both treatment groups and there was no significant difference in the appearance of gametocyte in both groups (RR 0.25, 95% CI 0.04 to 1.69; participants = 1474; studies = 4; I^2^ = 36%, Fig. 7) and (RR 0.43, 95% CI 0.11 to 1.65; participants = 599; studies = 1; I^2^ = 0%, Fig. 7).

### Anaemia

#### Mean haemoglobin (g/dL) at baseline

No significant difference was found in the mean haemoglobin (g/dL) at baseline in both treatment groups (MD 0.06, 95% CI − 0.07 to 0.18; participants = 2982; studies = 6; I^2^ = 0%, Additional file 8: S8).

#### Mean haemoglobin (g/dL) at Day 28 and 42

All five studies reported some measure of haematological recovery from baseline to day 28 in both treatment groups and no significant difference was found between the two groups in haematological recovery (Day 28, MD 0.04, 95% CI − 0.19 to 0.27; participants = 778; studies = 2; I^2^ = 0%, Additional file 8: S8). However, there was significant haematological recovery found among patients treated with DHA–PQ than AL at (Day 42, MD 0.35, 95% CI 0.12 to 0.59; participants = 1434; studies = 3; I^2^ = 35%, Additional file 8: S8).

### Adverse event

#### Gastrointestinal

**Fig. 8:**
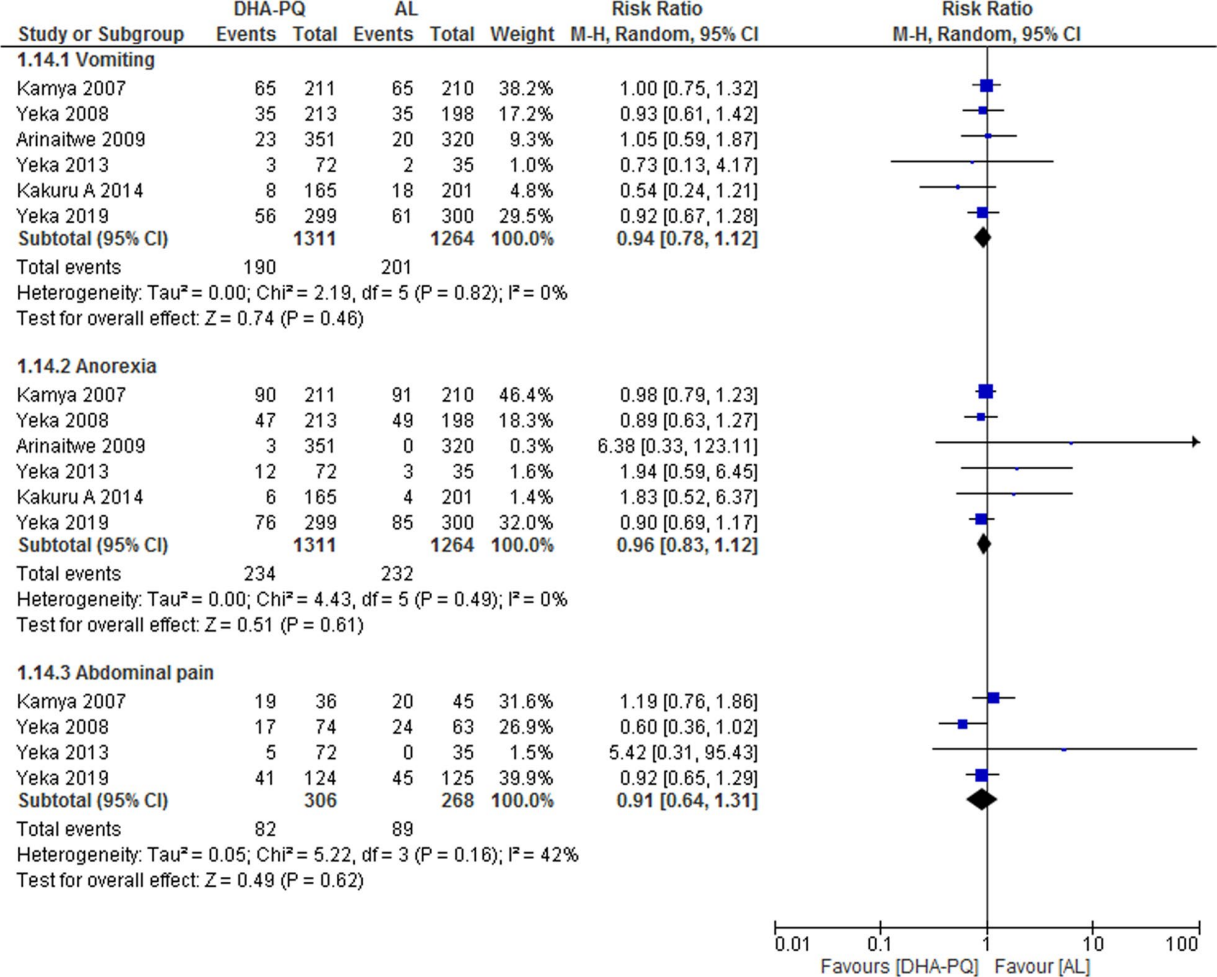
Forest plot of comparison: Dihydroartemisinin–piperaquine versus artemether–lumefantrine, outcome: other adverse events: Gastrointestinal

Studies reported vomiting, anorexia, and abdominal pain as an adverse event. However, no significant difference was found between the two intervention groups (RR 0.94, 95% CI 0.78 to 1.12; participants = 2575; studies = 6; I^2^ = 0%, Fig. 8), (RR 0.96, 95% CI 0.83 to 1.12; participants = 2575; studies = 6; I^2^ = 0%, Fig. 8), and (RR 0.91, 95% CI 0.64 to 1.31; participants = 574; studies = 4; I^2^ = 42%, Fig. 8).

#### Diarrhoea

**Fig. 9:**
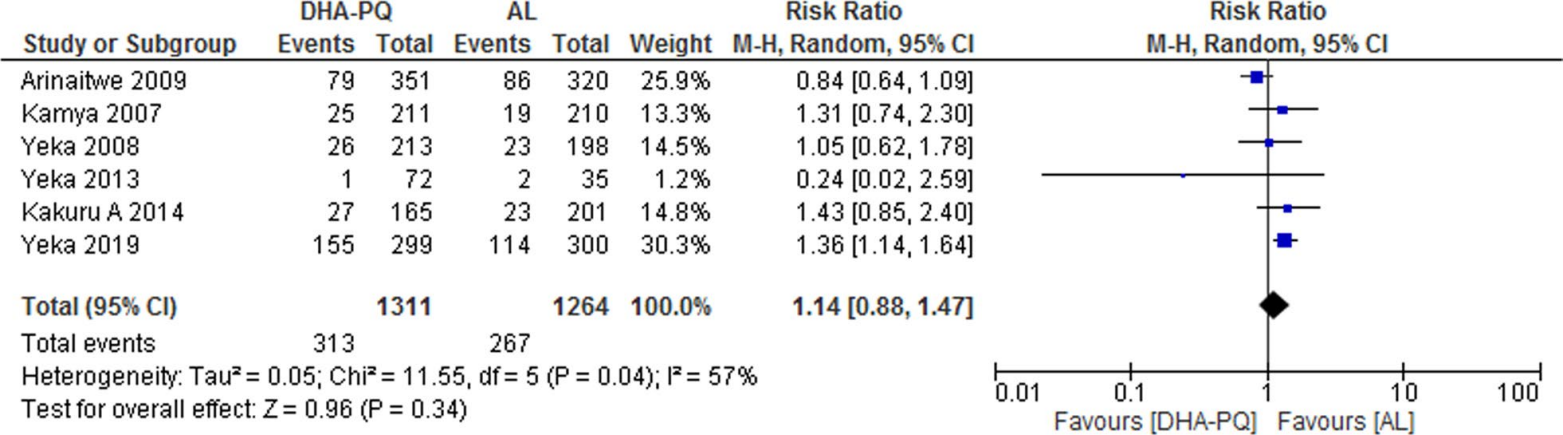
Forest plot of comparison: Dihydroartemisinin–piperaquine versus artemether–lumefantrine, outcome: other adverse events: Gastrointestinal (Diarrhoea)

Six studies reported diarrhoea as an adverse event. Diarrhoea was slightly more frequent in patients treated with DHA–PQ, but it was not statistically significant. Hence, there was no significant difference on the risk of diarrhoea in both treatment groups (RR 1.14, 95% CI 0.88 to 1.47; participants = 2575; studies = 6; I^2^ = 57%, high quality of evidence, Fig. 9).

In one study [42], considering 63 days of follow-up among all 837 treatments with study drugs; 415 adverse events due to cough (373 mild and 42 moderate severity), 179 adverse events due to diarrhea (168 mild, 10 moderate, and one severe), and 56 adverse events due to vomiting (all mild) were reported. Any adverse event due to cough, diarrhea, or vomiting occurred in 296 of 412 (72%) treatments with AL and 313 of 425 (74%) treatments with DHA–PQ. There were no statistically significant differences in the risks of these adverse events between the treatment arms for any time interval.

### Neuropsychiatric

**Fig. 10:**
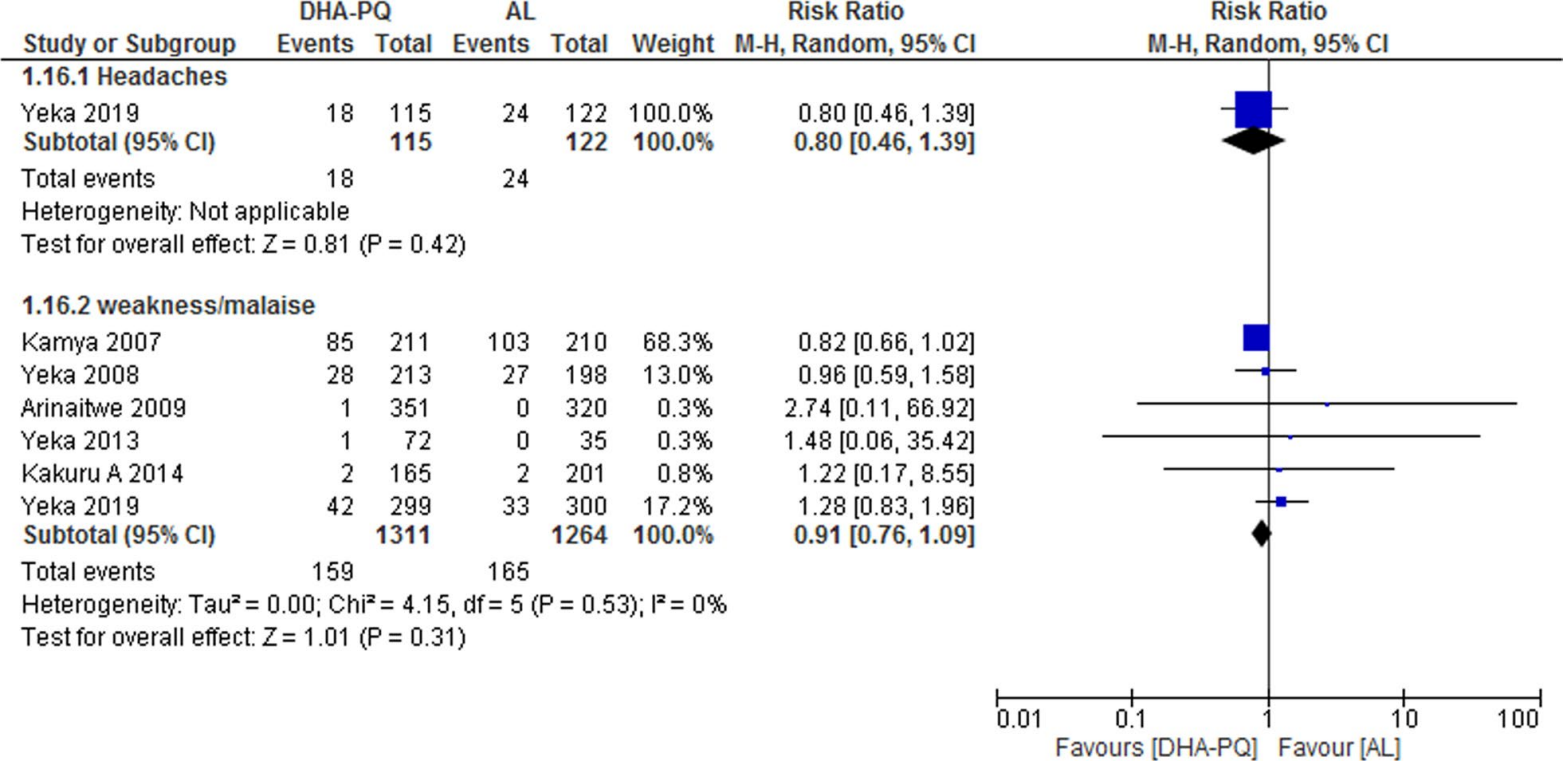
Forest plot of comparison: Dihydroartemisinin–piperaquine versus artemether–lumefantrine, outcome: other adverse events: Neuro-psychiatric

Studies reported headache and weakness or malaise as an adverse event and there was no significant difference between the two treatment groups (RR 0.80, 95% CI 0.46 to 1.39; participants = 237; studies = 1, Fig. 9) and (RR 0.91, 95% CI 0.76 to 1.09; participants = 2575; studies = 6; I^2^ = 0%, Fig. 10).

### Cardiorespiratory

**Fig. 11:**
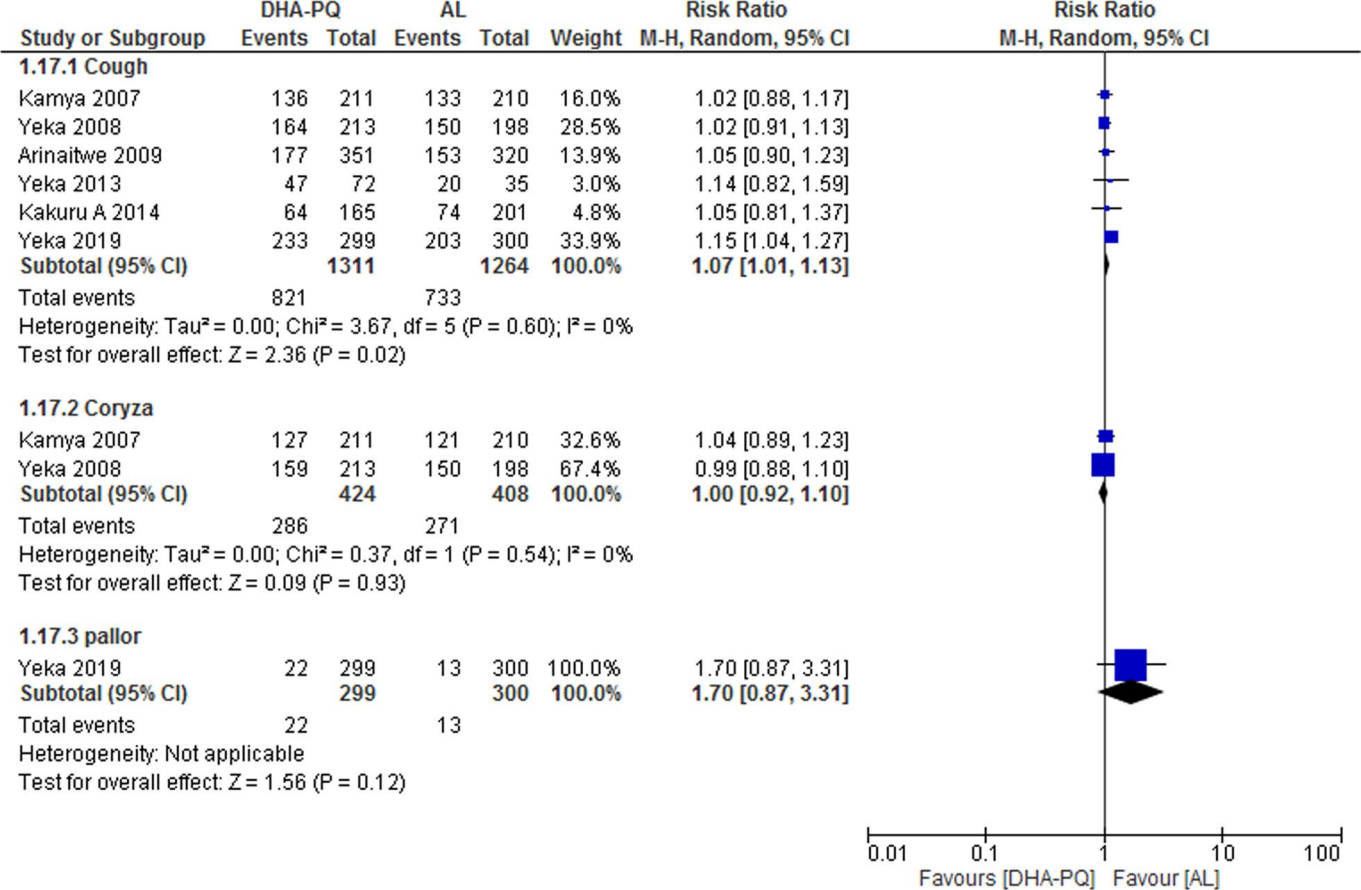
Forest plot of comparison: Dihydroartemisinin–piperaquine versus artemether–lumefantrine, outcome: other adverse events: Cardio-respiratory

Six studies reported cough as an adverse event. However, compared to AL, DHA–PQ was associated with a slightly higher frequency of cough (RR 1.07, 95% CI 1.01 to 1.13; participants = 2575; studies = 6; I^2^ = 0%, Fig. 11). In the other hand, studies reported that coryza and pallor were also slightly more frequent in patients treated with DHA–PQ than AL, but no significant difference have found between the two treatment group (RR 1.00, 95% CI 0.92 to 1.10; participants = 832; studies = 2; I^2^ = 0%, Fig. 11) and (RR 1.70, 95% CI 0.87 to 3.31; participants = 599; studies = 1*,* Fig. 11).

### Musculoskeletal/dermatological

**Fig. 12:**
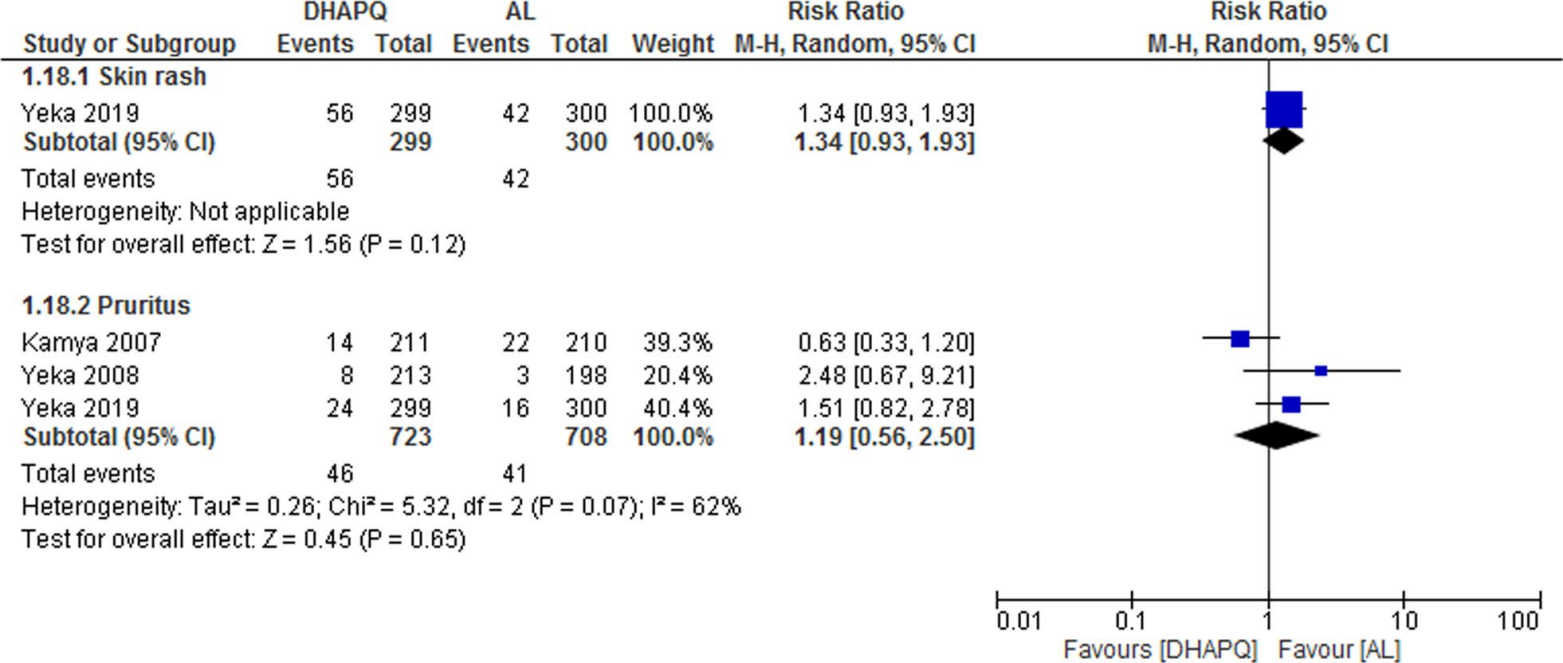
Forest plot of comparison: Dihydroartemisinin–piperaquine versus artemether–lumefantrine, outcome: other adverse events: Musculoskeletal/dermatological

Studies reported skin rash and pruritus as an adverse event and no significant difference was found between the two treatment groups (RR 1.34, 95% CI 0.93 to 1.93; participants = 599; studies = 1; I^2^ = 0%, Fig. 12) and (RR 1.19, 95% CI 0.56 to 2.50; participants = 1431; studies = 3; I^2^ = 62%, Fig. 12).

### Serious adverse event

**Fig. 13:**
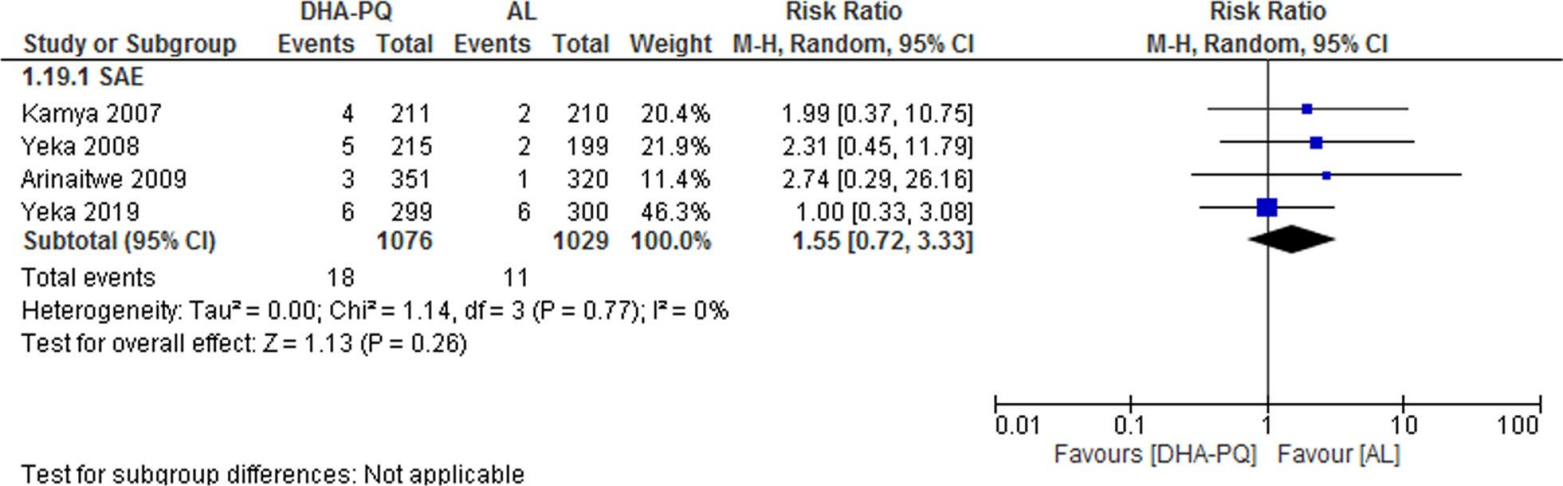
Forest plot of comparison: Dihydroartemisinin–piperaquine versus artemether–lumefantrine, outcome: Serious adverse events

Four studies reported 18 serious adverse events in DHA–PQ and 11 in the AL treatment group. However, the distributions of serious adverse events were not significantly different in the two treatment groups (RR 1.55, 95% CI 0.72 to 3.33; participants = 2105; studies = 4; I^2^ = 0%, Fig. 13). All serious adverse events were judged to be unrelated to study medications. No death has occurred in all studies. However, in one study [42] there were only five serious adverse events (two in the AL group and three in the DP group) and all were due to the development of severe anemia, which was likely a consequence of malaria and not the study drugs.

### Quality of the evidence

The quality of the evidence in this review was assessed using the GRADE approach and presented the evidence in six summary of findings tables for efficacy and safety (Summary of findings for the main comparison; Additional file 9: Additional Tables).

The evidence that DHA–PQ is more effective than AL at day 28, 42, and 63 unadjusted by genotyping was of *low,* moderate, and very low quality of evidence. There was considerable heterogeneity between studies at day 28 and 63. In addition, DHA–PQ consistently superiority over AL at day 42 adjusted by genotyping was of high quality of evidence and both DHA–PQ and AL performed better than the WHO standard of 5% PCR-adjusted treatment failure at day 28 in all trials (moderate quality of evidence). The quality of evidence was assessed on comparative adverse effects; cough slightly more frequent in DHA–PQ arm was of high quality of evidence. Generally, the quality of evidence of safety of the two treatments ranges from low to high quality.

## Discussion

### Summary of findings

This systematic review and meta-analysis focused on the safety and efficacy of DHA–PQ and AL for the treatment of uncomplicated falciparum malaria in children. The main finding of this meta-analysis is that the PCR unadjusted risk of recurrent falciparum parasitaemia at day 28 and 42 was significantly lower for participants treated with DHA–PQ than those treated with AL (low and moderate quality of evidence).

Early treatment failure was observed in three patients from the DHA–PQ group and 16 from the AL group had an early treatment failure in the three trials. At day 28, the PCR adjusted treatment failure was below 5% in both treatment arms without significant difference between the two treatment groups were observed (moderate quality of evidence). The PCR adjusted treatment failure day 42 was significantly lower for participants treated with DHA–PQ than those treated with AL (high quality of evidence). Nevertheless, at day 63 the PCR adjusted treatment failure in participants treated with AL was significantly lower than those who are treated with DHA–PQ (moderate quality of evidence).

The appearance of gametocyte at day 29–42 was significantly lower in patients treated with DHA–PQ than AL (moderate quality of evidence). In addition, In this review, most of the adverse events were mild or moderate severity and consistent with symptoms due to malaria. However, some adverse events like cough, anorexia, diarrhoea, and vomiting were the most common adverse events. In most studies, no significant difference was found in the proportion of study participants who experienced an adverse event of moderate and great severity between the DHA–PQ and AL treatment groups. But, cough was significantly more frequent in patients treated with DHA–PQ than AL (high quality of evidence).

### Public health implications

The observed high efficacy of DHA–PQ was similar to that of other studies conducted in Africa [43, 44] and a high transmission setting in Indonesia [45]. However, in a study done in Somalia, the recurrence of parasitaemia was lower in DHA–PQ as compared to AL arm [46] and after day 3, in both treatment groups none of the participants were parasitaemic. However, *Pfk13* non-synonymous mutations (R622I) with unknown impact on the parasite resistance phenotype have been seen at a very low rate.

Recent studies conducted in Mali, Somalia, Angola, and Papua New Guinea also reported that both DHA–PQ and AL were highly effective in the treatment of uncomplicated falciparum malaria [8, 47–49]. Similarly, a former review also reported that in Asia and Oceania, PCR-adjusted treatment failure at day 28 was similar between treatments [23]. However, one study conducted in Cambodia–Thailand border reported high recrudescence on DHA–PQ treatment group [50] and this might be related to artemisinin resistance in the sub-region [51, 52]. The reason for the rapid occurrence of DHA–PQ resistance in this sub-region unknown; however, pre-existing circulation of parasites resistant to artemisinin or PQ in this area was probably a major mediator for their evolution to multidrug resistance. Furthermore, two studies conducted in Angola in 2013 and 2015 reported PCR adjusted cure rate of 88% in AL at one site [53, 54]. This result is lower compared to other previous studies conducted in different sub-Saharan African countries [46, 55, 56]. Despite this, those patients who were enrolled in both Angolan studies took the evening doses of AL at home without any supervision, and the higher treatment failure rate might be explained by this.

Similarly, a recent study in Rwanda reported that 42 day PCR corrected efficacy was significantly better in patients with falciparum malaria treated with DHA–PQ [57]. Hence, Studies conducted in western Kenya and Mali reported that the risk of treatment failure in both group was below 5% at day 42 [44]. For interventions such as mass drug administrations or seasonal malaria prevention [58], an ACT which protects against subsequent infections, such as DHA–PQ [59], could play a crucial role.

In this review several studies using microscopic detection of gametocytes have shown no difference [17, 56, 60–62] and an increased risk of gametocyte detection after treatment with DHA–PQ [36, 43, 63]. Consequently, membrane-feeding experiments have confirmed that both microscopic and sub-patent gametocytaemia result in infectivity to mosquitoes, with a positive association between gametocyte density and mosquito infection rates [64]. Increasing age and recurrent parasitaemia were associated with an increased risk of first detection of gametocytes after therapy [65], but the estimated mean duration of gametocytaemia for children below 5, children from 5 to 9 and children 10 years and above was 9.4, 7.8, and 4.1 days, respectively [66]. Furthermore, one previous study have reported that prolonged gametocytaemia after treatment could be an early sign of the occurrence of drug resistance, which is also the case in the emergence of recrudescent infections [67].

In this systematic review, significant haematological recovery from the baseline has been observed among patients treated with AL than DHA–PQ at day 42. Recent studies in Africa have reported that there was significant haematological recovery from the baseline in both treatment groups [44, 56]. One study in Tanzania reported a significant increase in serum haemoglobin level in AL treatment arm at day 28 than DHA–PQ [55]. However, patients enrolled in one site had relatively high haemoglobin at baseline and maintained throughout the follow-up period. This might be related to the difference in nutritional status and other health conditions associated with anaemia, such as helminthic infections and concurrent infections [68–70]. Age difference could also be the reason for this [55]. Studies done elsewhere in Africa [55, 71, 72] reported that improvements in haemoglobin during follow-up could suggest that malaria might be a major causing factor to anaemia and the low haemoglobin levels at recruitment.

A recent study in Papua New Guinea reported a high frequency of cough without significant difference between the two treatment groups [47]. A former study conducted in Zambia reported a high frequency of cough in DHA–PQ group than AL [18]. A study on AL and DHA–PQ safety and tolerability reported cough, diarrhoea, vomiting, and anaemia as the most commonly reported adverse events [42]. In a review done in Asia, gastrointestinal complaints were the most common adverse events associated with DHA–PQ, with no evidence of severe drug toxicity [73] and recent study in Africa also reported vomiting as a common adverse event in patients treated with DHA–PQ [74]. In breastfeeding infants DHA–PQ has previously been linked to an increased risk of vomiting [75]. The mechanism accountable for the increased risk of early vomiting among breastfeeding participants treated with DHA–PQ is not known. However, the temporal relationship suggests that the susceptibility of gastric mucosa of breastfed infants could be related to the pro-emetic effect of piperaquine than that in weaned infants [75]. To determine whether the co-administered milk may also affect this interaction further assessment might be needed. However, the absence of effect with AL implies that the mechanism is given to DHA–PQ, most likely piperaquine.

In this systematic review, four studies reported 21 serious adverse events in DHA–PQ and 13 in the AL treatment group. However, the distributions of serious adverse events were not significantly different in the two treatment groups. All serious adverse events were not related to study medications. No death has occurred in any of the studies. This might be justified by the fact that these studies were conducted among participants with uncomplicated malaria rather than the severe form which can lead to death.

Treatment failure could be occurred due to the drug’s ineffectiveness or development of resistance, as it may be due to insufficient drug levels [65]. Furthermore, treatment failure may occur due to resistance, sub-therapeutic levels that may occur due to non-adherence, or inadequate absorption. To identify risk factors for treatment failure further studies should be conducted. Also, further trials with detailed descriptions of patients’ characteristics with recrudescence are also very important. Besides, to investigate the association of AL and DHA–PQ resistance in the places where *P. falciparum* is endemic molecular surveillance may also play an important role in detecting genetic markers.

### Study limitations

The study has some limitations. A majority of included studies were conducted in Tororo District Eastern region Uganda where malaria transmission intensity is high. The result of this study might not be representative of other regions in Uganda where malaria transmission intensity is low and moderate. Most studies reported treatment failure at 28 and 42 days, this review might not provide strong evidence about the long-term post-treatment prophylactic effect of the two drugs.

## Conclusion

This systematic review provides comprehensive evidence about the treatment efficacy and safety of ACT in children in an area of malaria-endemic areas in Uganda. The overall parasite clearance, drug efficacy, and safety were good enough. Compared to AL, DHA–PQ appeared to reduce treatment failure and gametocyte carriage in Ugandan children. This may trigger DHA–PQ to become the first-line treatment option. Both treatments were safe and tolerable. As ACT resistance is emerging in different parts of the world, continuous studies that measure the efficacy of DHA–PQ and AL with 42 and 63 days follow-up are needed.

## Supplementary Information

**Additional file 1: S1.** Forest plot of comparison: Dihydroartemisinin-piperaquine versus artemether-lumefantrine, outcome: PCR-unadjusted treatment failures at day 63.

**Additional file 2: S2.** Forest plot of comparison: Dihydroartemisinin-piperaquine versus artemether-lumefantrine, outcome: PCR-adjusted treatment failures at day 63.

**Additional file 3: S3.** Forest plot of comparison: Dihydroartemisinin-piperaquine versus artemether-lumefantrine, outcome: Fever clearances on day 1.

**Additional file 4: S4.** Forest plot of comparison: Dihydroartemisinin-piperaquine versus artemether-lumefantrine, outcome: Fever clearances on day 2.

**Additional file 5: S5.** Forest plot of comparison: Dihydroartemisinin-piperaquine versus artemether-lumefantrine, outcome: Fever clearances on day 3.

**Additional file 6: S6.** Forest plot of comparison: Dihydroartemisinin-piperaquine versus artemether-lumefantrine, outcome: Parasite clearances.

**Additional file 7: S7.** Forest plot of comparison: Dihydroartemisinin-piperaquine versus artemether-lumefantrine, outcome: Gametocyte carriages at baseline.

**Additional file 8: S8.** Forest plot of comparison: Dihydroartemisinin-piperaquine versus artemether-lumefantrine, outcome: Anemia.

**Additional file 9.** Additional Tables: GRADE Summary of finding tables.

## Data Availability

All relevant data are within the manuscript and its additional information files.

## Abbreviations

ACT: Artemisinin-based combination therapy
ACPR: Adequate clinical and parasitological response
AL: Artemether–lumefantrine
BW: Body weight
CENTRAL: Cochrane Central Register of Controlled Trials
CI: Confidence interval
CM: Complicated malaria
DHA–PQ: Dihydroartemisinin–piperaquine
DHS: Demographic Health Survey
ETF: Early treatment failure
GADE: Grading of Recommendations, Assessment, Development, and Evaluations
Hgb: Haemoglobin
LCT: Late clinical failure
LPF: Late parasitological failure
PCR: Polymerase chain reaction
PICO: Population, intervention, comparison, and outcome
PRISMA: Preferred Reporting Items for Systematic Reviews and Meta-Analyses
RDT: Rapid diagnostic test
SAE: Serious adverse event
SD: Standard deviation
WHO: World Health Organization
