# Supplementary figures and images for: Efficacy and safety of dihydroartemisinin–piperaquine versus artemether–lumefantrine for treatment of uncomplicated Plasmodium falciparum malaria in Ugandan children: a systematic review and meta-analysis of randomized control trials
Source: Malar J. 2021 Apr 1;20:174. doi: 10.1186/s12936-021-03711-4 (PMC8017896; doi:10.1186/s12936-021-03711-4)

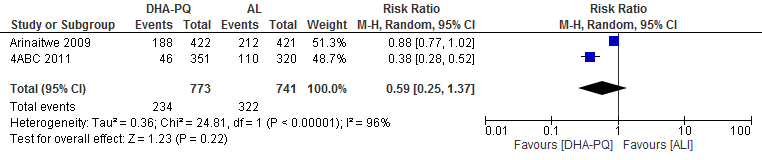

Supplement: Supplementary file 1 — Additional file 1: S1. Forest plot of comparison: Dihydroartemisinin-piperaquine versus artemether-lumefantrine, outcome: PCR-unadjusted treatment failures at day 63. [file 12936_2021_3711_MOESM1_ESM.docx]

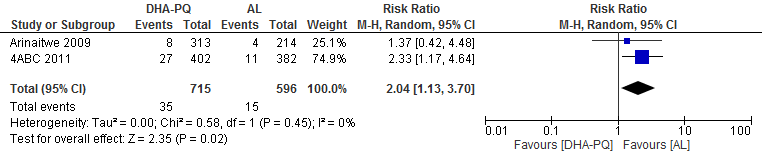

Supplement: Supplementary file 2 — Additional file 2: S2. Forest plot of comparison: Dihydroartemisinin-piperaquine versus artemether-lumefantrine, outcome: PCR-adjusted treatment failures at day 63. [file 12936_2021_3711_MOESM2_ESM.docx]

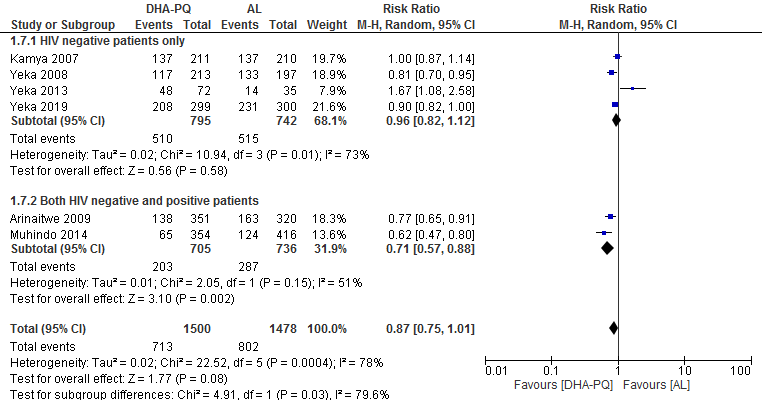

Supplement: Supplementary file 3 — Additional file 3: S3. Forest plot of comparison: Dihydroartemisinin-piperaquine versus artemether-lumefantrine, outcome: Fever clearances on day 1. [file 12936_2021_3711_MOESM3_ESM.docx]

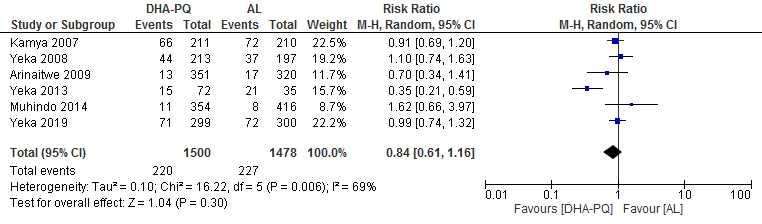

Supplement: Supplementary file 4 — Additional file 4: S4. Forest plot of comparison: Dihydroartemisinin-piperaquine versus artemether-lumefantrine, outcome: Fever clearances on day 2. [file 12936_2021_3711_MOESM4_ESM.docx]

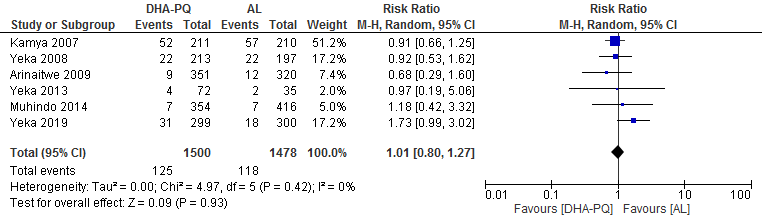

Supplement: Supplementary file 5 — Additional file 5: S5. Forest plot of comparison: Dihydroartemisinin-piperaquine versus artemether-lumefantrine, outcome: Fever clearances on day 3. [file 12936_2021_3711_MOESM5_ESM.docx]

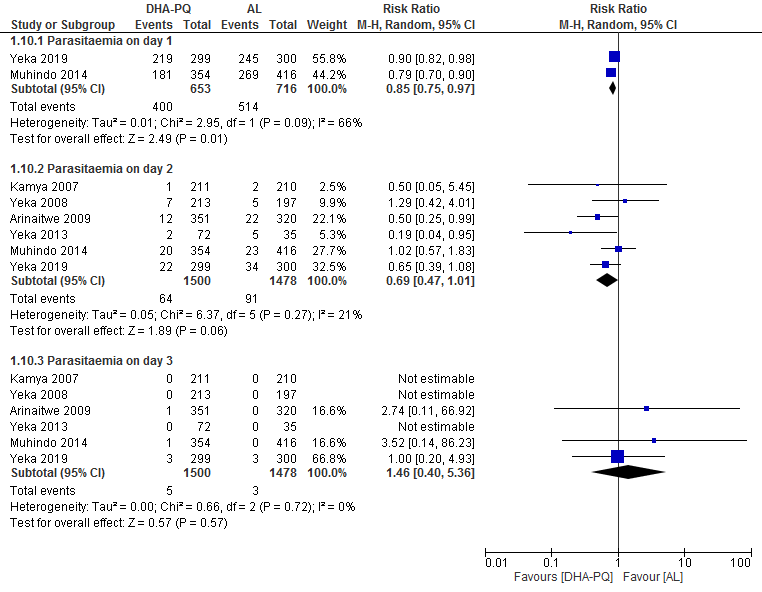

Supplement: Supplementary file 6 — Additional file 6: S6. Forest plot of comparison: Dihydroartemisinin-piperaquine versus artemether-lumefantrine, outcome: Parasite clearances. [file 12936_2021_3711_MOESM6_ESM.docx]

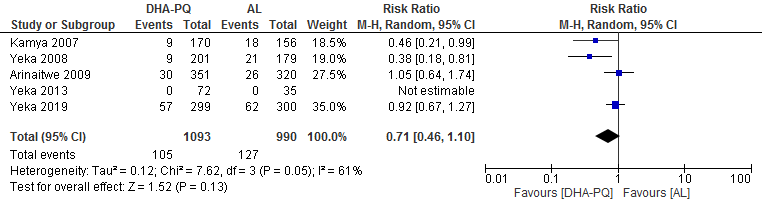

Supplement: Supplementary file 7 — Additional file 7: S7. Forest plot of comparison: Dihydroartemisinin-piperaquine versus artemether-lumefantrine, outcome: Gametocyte carriages at baseline. [file 12936_2021_3711_MOESM7_ESM.docx]

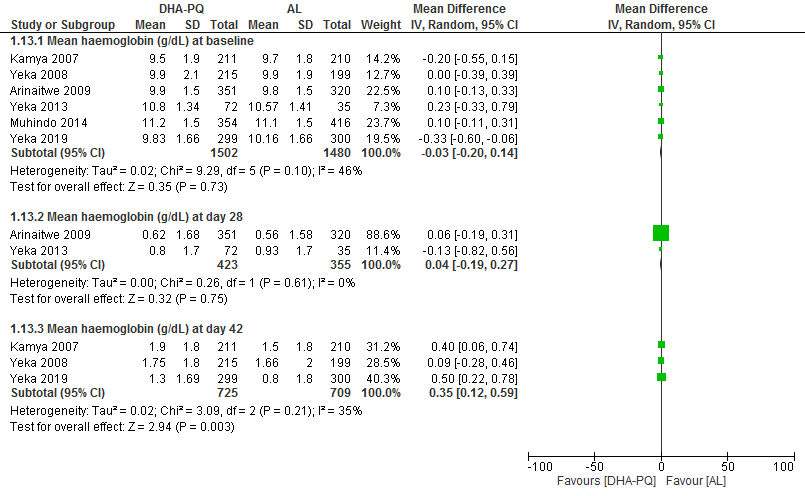

Supplement: Supplementary file 8 — Additional file 8: S8. Forest plot of comparison: Dihydroartemisinin-piperaquine versus artemether-lumefantrine, outcome: Anemia. [file 12936_2021_3711_MOESM8_ESM.docx]
