# Supplementary material for: Efficacy and safety of dihydroartemisinin–piperaquine versus artemether–lumefantrine for treatment of uncomplicated Plasmodium falciparum malaria in Ugandan children: a systematic review and meta-analysis of randomized control trials
Source: Malar J. 2021 Apr 1;20:174. doi: 10.1186/s12936-021-03711-4 (PMC8017896; doi:10.1186/s12936-021-03711-4)
Supplement: Supplementary file 9 — Additional file 9. Additional Tables: GRADE Summary of finding tables. [file 12936_2021_3711_MOESM9_ESM.docx]

## GRADE Summary of finding table

**Table S1: Summary of finding (Treatment failure)**

| **Dihydroartemisinin-piperaquine compared to Artemether-lumefantrine for the treatment of uncomplicated *falciparum* malaria in Ugandan children** | | | | | | |
| --- | --- | --- | --- | --- | --- | --- |
| **Patient or population**: **The treatment of uncomplicated *falciparum* malaria in Ugandan children**  **Setting**: **Malaria endemic settings in Uganda**  **Intervention**: **Dihydroartemisinin piperaaquine**  **Comparison**: **Artemether-lumefantrine** | | | | | | |
| Outcomes | **Anticipated absolute effects^*^** (95% CI) | | Relative effect (95% CI) | № of participants  (studies) | Certainty of the evidence (GRADE) | Comments |
|  | **Risk with Artemether-Lumefantrine** | **Risk with Dihydroartemisinin Piperaquine** |  |  |  |  |
| PCR-unadjusted treatment failure at day 28 | 459 per 1,000 | **138 per 1,000** (87 to 225) | **RR 0.30** (0.19 to 0.49) | 7863 (5 RCTs) | **⨁⨁◯◯ LOW ^c,d^** |  |
| PCR-adjusted treatment failure at day 28 | 2 per 100 | **2 per 100** (1 to 3) | **RR 0.70** (0.40 to 1.23) | 2411 (5 RCTs) | **⨁⨁⨁◯ MODERATE ^a,e,f^** |  |
| PCR-unadjusted treatment failure at day 42 | 417 per 1,000 | **221 per 1,000** (158 to 317) | **RR 0.53** (0.38 to 0.76) | 1618 (4 RCTs) | **⨁⨁⨁◯ MODERATE ^d,e^** |  |
| PCR-adjusted treatment failure at day 42. | 9 per 100 | **4 per 100** (2 to 6) | **RR 0.45** (0.28 to 0.72) | 1370 (5 RCTs) | **⨁⨁⨁⨁ HIGH ^e^** |  |
| PCR-unadjusted treatment failure at day 63. | 435 per 1,000 | **256 per 1,000** (109 to 595) | **RR 0.59** (0.25 to 1.37) | 1514 (2 RCTs) | **⨁◯◯◯ VERY LOW ^b,d,g^** |  |
| PCR-adjusted treatment failure at day 63. | 3 per 100 | **5 per 100** (3 to 9) | **RR 2.04** (1.13 to 3.70) | 1311 (2 RCTs) | **⨁⨁⨁◯ MODERATE ^g^** |  |
| ***The risk in the intervention group** (and its 95% confidence interval) is based on the assumed risk in the comparison group and the **relative effect** of the intervention (and its 95% CI).  **CI:** Confidence interval; **RR:** Risk ratio; **MD:** Mean difference | | | | | | |
| **GRADE Working Group grades of evidence** **High certainty:** We are very confident that the true effect lies close to that of the estimate of the effect **Moderate certainty:** We are moderately confident in the effect estimate: The true effect is likely to be close to the estimate of the effect, but there is a possibility that it is substantially different **Low certainty:** Our confidence in the effect estimate is limited: The true effect may be substantially different from the estimate of the effect **Very low certainty:** We have very little confidence in the effect estimate: The true effect is likely to be substantially different from the estimate of effect | | | | | | |

#### Explanations

a. Both the sample size and treatment effects are large.

b. No significant difference was found between the two intervention groups.

c. Three trials with a high risk of bias.

d. The statistical heterogeneity was high.

e. Studies have low risk of bias. Exclusion of studies with a high risk of bias doesn't change the result.

f. The 95% CI include appreciable benefit or harm but RR was below 0.75 and OIS criteria were met

g. One of the studies was open label.

Table S2: Summary of finding (Adverse event and serious adverse event)

| **Dihydroartemisinin piperaquine compared to artemether-lumefantrine for the treatment of uncomplicated *falciparum* malaria in Ugandan children** | | | | | | |
| --- | --- | --- | --- | --- | --- | --- |
| **Patient or population: the treatment of uncomplicated *falciparum* malaria in Ugandan children**  **Setting: Malaria endemic settings in Uganda**  **Intervention: Dihydroartemisinin-piperaquine**  **Comparison: Artemether-lumefantrine** | | | | | | |
| Outcomes | **Anticipated absolute effects^*^** (95% CI) | | Relative effect (95% CI) | № of participants  (studies) | Certainty of the evidence (GRADE) | Comments |
|  | **Risk with artemether-lumefantrine** | **Risk with dihydroartemisinin piperaquine** |  |  |  |  |
| Other adverse events: Gastrointestinal - Vomiting | 16 per 100 | **15 per 100** (12 to 18) | **RR 0.94** (0.78 to 1.12) | 2575 (6 RCTs) | **⨁⨁⨁⨁ HIGH ^a,g^** |  |
| Other adverse events: Gastrointestinal - Anorexia | 18 per 100 | **18 per 100** (15 to 21) | **RR 0.96** (0.83 to 1.12) | 2575 (6 RCTs) | **⨁⨁⨁⨁ HIGH ^a,g^** |  |
| Other adverse events: Gastrointestinal - Abdominal pain | 33 per 100 | **30 per 100** (21 to 44) | **RR 0.91** (0.64 to 1.31) | 574 (4 RCTs) | **⨁⨁⨁◯ MODERATE ^f^** |  |
| Other adverse events: Gastrointestinal (Diarrhoea) | 211 per 1,000 | **241 per 1,000** (186 to 311) | **RR 1.14** (0.88 to 1.47) | 2575 (6 RCTs) | **⨁⨁⨁◯ MODERATE ^e,f,h^** |  |
| Other adverse events: Neuro-psychiatric - Headaches | 20 per 100 | **16 per 100** (9 to 27) | **RR 0.80** (0.46 to 1.39) | 237 (1 RCT) | **⨁⨁⨁◯ MODERATE ^d,f^** |  |
| Other adverse events: Neuro-psychiatric - weakness/malaise | 13 per 100 | **12 per 100** (10 to 14) | **RR 0.91** (0.76 to 1.09) | 2575 (6 RCTs) | **⨁⨁⨁⨁ HIGH ^a,g^** |  |
| Other adverse events: Cardio-respiratory - Cough | 58 per 100 | **62 per 100** (59 to 66) | **RR 1.07** (1.01 to 1.13) | 2575 (6 RCTs) | **⨁⨁⨁⨁ HIGH ^g^** |  |
| Other adverse events: Cardio-respiratory - Coryza | 66 per 100 | **66 per 100** (61 to 73) | **RR 1.00** (0.92 to 1.10) | 832 (2 RCTs) | **⨁⨁⨁◯ MODERATE ^d,f^** |  |
| Other adverse events: Cardio-respiratory - pallor | 4 per 100 | **7 per 100** (4 to 14) | **RR 1.70** (0.87 to 3.31) | 599 (1 RCT) | **⨁⨁⨁◯ MODERATE ^c^** |  |
| Other adverse events: Musculoskeletal/dermatological - Skin rash | 14 per 100 | **19 per 100** (13 to 27) | **RR 1.34** (0.93 to 1.93) | 599 (1 RCT) | **⨁⨁⨁◯ MODERATE ^f^** |  |
| Other adverse events: Musculoskeletal/dermatological - Pruritus | 6 per 100 | **7 per 100** (3 to 14) | **RR 1.19** (0.56 to 2.50) | 1431 (3 RCTs) | **⨁⨁⨁◯ MODERATE ^c^** |  |
| Serious Adverse Events - SAE | 1 per 100 | **2 per 100** (1 to 4) | **RR 1.55** (0.72 to 3.33) | 2105 (4 RCTs) | **⨁⨁⨁◯ MODERATE ^c^** |  |
| ***The risk in the intervention group** (and its 95% confidence interval) is based on the assumed risk in the comparison group and the **relative effect** of the intervention (and its 95% CI).  **CI:** Confidence interval; **RR:** Risk ratio; **MD:** Mean difference | | | | | | |
| **GRADE Working Group grades of evidence** **High certainty:** We are very confident that the true effect lies close to that of the estimate of the effect **Moderate certainty:** We are moderately confident in the effect estimate: The true effect is likely to be close to the estimate of the effect, but there is a possibility that it is substantially different **Low certainty:** Our confidence in the effect estimate is limited: The true effect may be substantially different from the estimate of the effect **Very low certainty:** We have very little confidence in the effect estimate: The true effect is likely to be substantially different from the estimate of effect | | | | | | |

#### Explanations

a. The point estimate is between 0.75 and 1.25. we don't rate down for imprecision and OIS criteria are met.

b. No blinding of outcome assessors.

c. There 95% CI is wide and no significant difference was found between two intervention groups.

d. Few participants or events.

e. No blinding of outcome assessors. The exclusion of studies with a high risk of bias did not change the result.

f. No significant difference was found between the two intervention groups.

g. No blinding of outcome assessors and unclear risk of bias. The exclusion of studies with a high risk of bias did not change the result.

h. The cause heterogeneity was explained by sub-group analysis.
